# Demystifying Activity Origin of M–N–C Single‐Atomic Mediators Toward Expedited Rate‐Determining Step in Li–S Electrochemistry

**DOI:** 10.1002/smsc.202200059

**Published:** 2022-08-21

**Authors:** Jia Jin, Zhongti Sun, Tianran Yan, Zixiong Shi, Meiyu Wang, Ting Huang, Yifan Ding, Jingsheng Cai, Peng Wang, Liang Zhang, Jingyu Sun

**Affiliations:** ^1^ College of Energy, Soochow Institute for Energy and Materials InnovationS (SIEMIS) Light Industry Institute of Electrochemical Power Sources Jiangsu Provincial Key Laboratory for Advanced Carbon Materials and Wearable Energy Technologies Soochow University Suzhou 215006 P. R. China; ^2^ School of Materials Science and Engineering Jiangsu University Zhenjiang 212013 P. R. China; ^3^ Institute of Functional Nano & Soft Materials (FUNSOM) Jiangsu Provincial Key Laboratory for Carbon-Based Functional Materials and Devices Soochow University Suzhou 215006 P. R. China; ^4^ Materials Science and Engineering Physical Science and Engineering Division King Abdullah University of Science and Technology (KAUST) Thuwal 23955-6900 Saudi Arabia; ^5^ College of Engineering and Applied Sciences and Collaborative Innovation Center of Advanced Microstructures National Laboratory of Solid-State Microstructures Jiangsu Key Laboratory of Artificial Functional Materials Nanjing University Nanjing 210093 P. R. China

**Keywords:** Li–S batteries, M–N–C, rate-determining step, single-atomic mediator, sulfur reduction reaction

## Abstract

Sluggish sulfur reduction reaction (SRR) kinetics remains a formidable challenge in Li–S electrochemistry. In this sense, the rational design of single‐atom species has become a burgeoning practice to expedite sulfur redox, where the underlying catalytic mechanism otherwise remains elusive. Herein, a class of metal single‐atom modified porous carbon nanofiber films (MSA PCNFs, M = Fe, Co, or Ni), fabricated via a generic synthetic strategy, as mediators to boost SRR kinetics is reported. Throughout electrokinetic measurement and operando instrumental probing, NiSA PCNF is evidenced to harness the catalytic superiority toward the rate‐determining step (i.e., liquid–solid conversion) of the SRR process. Density functional theory (DFT) simulations further reveal that the catalytic features of M–N–C moieties in catalyzing the Li_2_S precipitation rely heavily upon the coordination environments of adjacent carbon atoms and *d*‐orbital configurations of metal centers. In response, the thus‐derived S/NiSA PCNF cathode realizes an encouraging areal capacity of 14.12 mAh cm^−2^ under elevated sulfur loading (10.2 mg cm^−2^) and lean electrolyte usage (E/S ratio ≈ 5.5 μL mg^−1^). This work offers insight into the identification of exact catalytic moieties for different transition metal M–N–C single‐atom SRR mediators, showcasing a meaningful guidance and potential impact on Li–S catalysis.

## Graphical abstract

A class of metal single‐atom modified porous carbon nanofiber films (MSA PCNFs, M = Fe, Co, or Ni) is synthesized, where NiSA PCNF is found to harness a superior catalytic effect to expedite the Li_2_S precipitation process. The thus‐derived Li–S batteries achieve an encouraging areal capacity of 14.12 mAh cm^−2^ based on S/NiSA PCNF cathode.

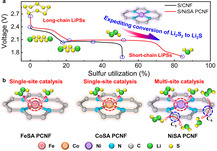

## 1 Introduction

The sluggish charge transfer kinetics and multi‐step conversion of S_8_ molecules into Li_2_S during sulfur reduction reaction (SRR) inevitably result in limited sulfur utilization, poor rate capability, and short cycle life, thereby greatly hindering the practical application of Li–S batteries.^[^ ^1^, ^2^, ^3^, ^4^, ^5^, ^6^, ^7^, ^8^ ^]^ As for the sulfur cathode, it undergoes a typical SRR process throughout solid (S_8_)‐liquid (soluble polysulfides)‐solid (insoluble Li_2_S_2_)‐solid (insoluble Li_2_S) phase transformation upon discharge. In this sense, S_8_ is first reduced through a sequence of lithium polysulfides to Li_2_S_4_, which delivers 25% of the theoretical capacity. The further reduction of Li_2_S_4_ to Li_2_S, marked as the rate‐determining step, contributes to the other 75% of the theoretical capacity.^[^ ^9^ ^]^ Note that the conversion of sulfur is significantly handicapped by the depression of solid‐state diffusion, accordingly inducing the premature end of discharge, which leads to a conspicuous difference between the actual capacity and the theoretical value. This phenomenon would become more rampant under elevated sulfur loading conditions. Therefore, designing efficient electrocatalysts to expedite the liquid–solid transformation from Li_2_S_4_ to Li_2_S is strategically meaningful and imperative.

To date, versatile polar metal compounds with ultrafine nanostructures have been widely explored as electrocatalyst candidates targeting the acceleration of Li_2_S_4_ → Li_2_S conversion.^[^ ^10^, ^11^ ^]^ Despite the fruitful progress, the fundamentally catalytic insights of Li_2_S precipitation during SRR remain rather elusive. Meanwhile, the application of such bulk metal‐based architectures in the cathode will inevitably reduce the loading ratio of sulfur, which is highly likely to undermine the energy density of the full cells. Along this line, the development of lightweight electrocatalysts affording excellent electrical conductivity and sufficient catalytic activity is desirable to realize the full potential of a sulfur electrode.^[^ ^12^ ^]^

Single‐atom catalysts (SACs) with atomically dispersed metals stabilized on a suitable support have attracted intensive interest due to their impressive catalytic capability and maximized atom utilization efficiency.^[^ ^13^, ^14^, ^15^, ^16^, ^17^, ^18^, ^19^, ^20^ ^]^ In the Li–S realm, transition‐metal‐derived SACs harnessing representative M–N–C configurations (e.g., MN_4_C_4_) have readily demonstrated encouraging performances in boosting sulfur electrochemistry.^[^ ^21^, ^22^, ^23^ ^]^ Nevertheless, unambiguous identification of exact catalytic moieties in different metal‐centered M–N–C SRR electrocatalysts is still lacking. In further contexts, a definitive structure–property correlation in common SACs employed as SRR electrocatalysts has not yet been established.

Herein, we report a universal route to derive representative non‐precious metal single‐atom modified porous carbon nanofiber films (MSA PCNFs, M = Fe, Co, or Ni) as electrocatalytic hosts to dictate SRR kinetics. The atomistic architectures and coordination configurations of prepared MSA (MN_4_C_4_) PCNFs are exhaustively identified by high‐angle annular dark field scanning transmission electron microscope (HAADF‐STEM) in combination with X‐Ray absorption near‐edge structure (XANES) and extended X‐Ray absorption fine structure (EXAFS). Density functional theory (DFT) calculation is employed to predict the distinctive catalytic properties of MSA PCNFs toward the Li_2_S precipitation, indicating that the mechanistic pathways rely not only upon a metallic single‐atomic center but also neighboring carbon environment. In this regard, MSA PCNFs as efficient electrocatalytic hosts follow the activity order of NiSA PCNF > CoSA PCNF > FeSA PCNF, which is further corroborated by electrokinetic measurements and electrochemical performance evaluations.

## 2 Results and Discussion

**Figure 1:**
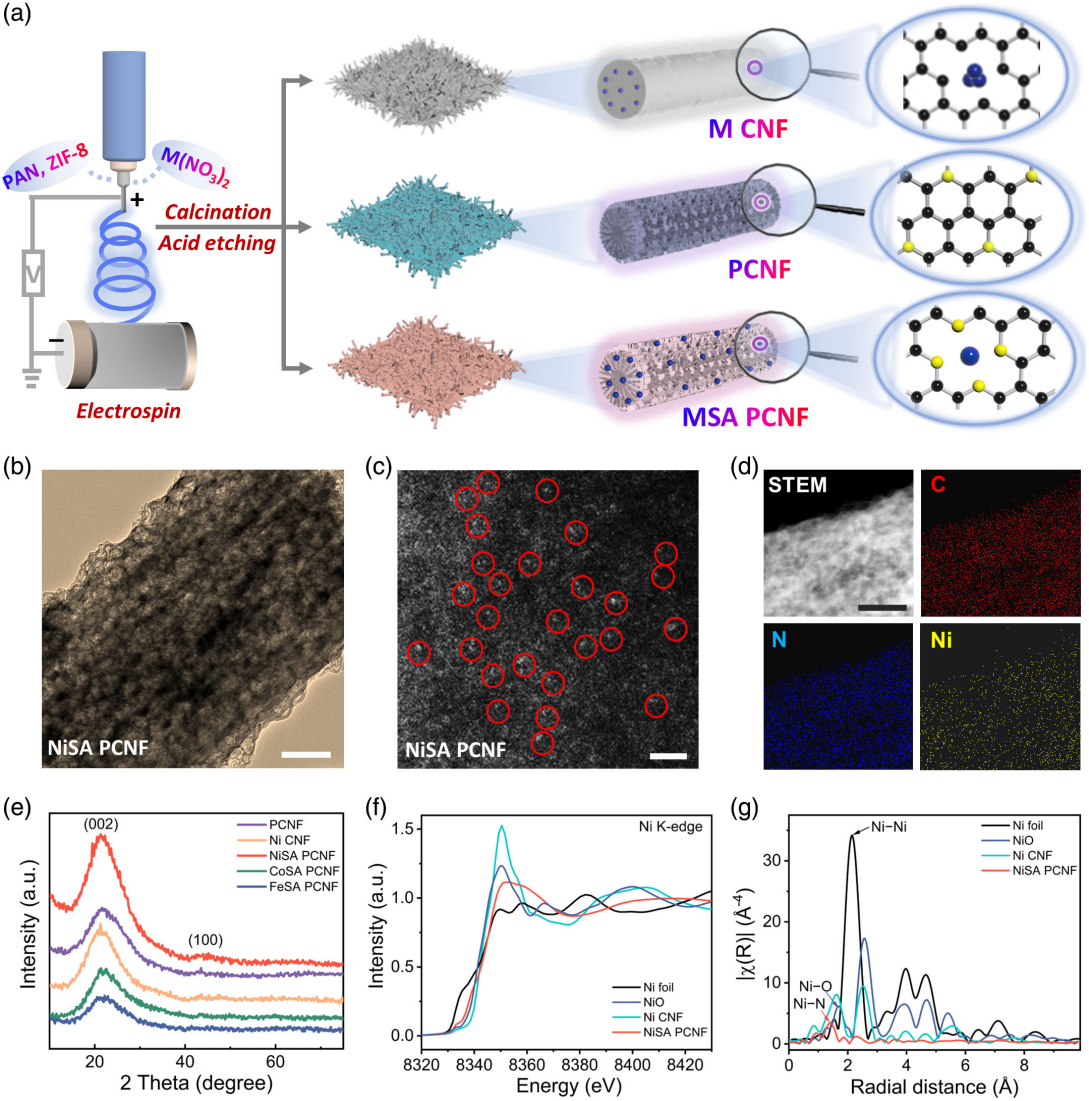
Synthesis and characterization of MSA (MN_4_C_4_) porous carbon nanofiber film (PCNF). a) Schematic illustration of the fabrication process of MSA PCNF. b) Transmission electron microscopy (TEM) image of NiSA PCNF. c) High‐angle annular dark field scanning transmission electron microscopy (HAADF‐STEM) image of NiSA PCNF. d) Energy‐dispersive X‐Ray spectroscopy (EDS) maps of NiSA PCNF. e) X‐Ray diffraction (XRD) patterns of NiSA PCNF, CoSA PCNF, FeSA PCNF, PCNF, and Ni CNF. f) X‐Ray absorption near‐edge structure (XANES) of NiSA PCNF, Ni CNF, Ni foil, and NiO. g) Fourier‐transformed k3‐weighted *χ*(k) function of the EXAFS (FT‐EXAFS spectra in *R* space of NiSA PCNF, Ni CNF, Ni foil, and NiO. Scar bars: b) 100 nm. c) 2 nm. d) 100 nm.

The generic fabrication of MSA PCNF affording dispersed metal single‐atoms deals with a two‐step procedure (**Figure** **1**a). First, a mixed solution containing zeolite imidazolate framework 8 (ZIF‐8) and M(NO_3_)_2_·6H_2_O was impregnated into a polyacrylonitrile (PAN) host by a typical electrospinning method to form an M^2+^/PAN/ZIF‐8 fibrous composite. Upon thorough drying, such a composite was subject to thermal annealing at 800 °C in an Ar atmosphere to derive MSA PCNF. This synthetic strategy can be readily generalized to commonly non‐precious metal elements (Fe, Co, or Ni in this case). It is also anticipated to be applicable for the scalable production of M–N–C SACs frameworks with cost‐effective features. Note that M CNF or bare PCNF can also be acquired by adopting a similar synthesis strategy merely without the employment of ZIF‐8 or M(NO_3_)_2_·6H_2_O precursor. The thus‐produced MSA PCNF membrane shows excellent flexibility and mechanical stability on a macroscopic scale, which could remain intact under different bending states (Figure S1, Supporting Information). Extensive transmission electron microscope (TEM) characterizations were performed to inspect detailed morphologies of MSA PCNFs (Figure S2, Supporting Information). As shown in the typical TEM image in Figure 1b, representative NASA PCNF possesses 3D interconnected architecture with uniformly distributed pores throughout the nanofiber, which is beneficial to mass transport during SRR catalytic process. This is in stark contrast to the control sample (Ni CNF) which lacks conspicuous pores (Figure S3, Supporting Information). High‐resolution TEM (HRTEM) examination over synthesized NiSA PCNF reveals the presence of ample holes and the absence of metal clusters (Figure S4, Supporting Information). The atomically dispersed feature of MSA PCNF was ultimately demonstrated by HAADF‐STEM imaging. As displayed in Figure 1c and S5, Supporting Information, Ni, Co, or Fe single atoms are obviously witnessed as bright spots highlighted by red circles.^[^ ^24^ ^]^ The metal loading within MSA PCNFs was determined by inductively coupled plasma atomic emission spectroscopy (ICP‐AES), which reveals similar metal contents of ≈0.5 at% (Table S1, Supporting Information).

Elemental characterization by energy‐dispersive X‐Ray spectroscopy (EDS) mapping under a STEM mode indicates that C, N, and Ni are homogeneously distributed over NiSA PCNF (Figure 1d). The X‐Ray diffraction (XRD) patterns of MSA PCNF, Ni CNF, and bare PCNF all manifest two broad peaks centered at 26.2° and 44.0° (Figure 1e), which can be indexed as featured carbon (002) and (100) signals, respectively. Note that diffraction peaks corresponding to metallic and oxidized forms of Ni are absent in these XRD patterns. Compositional analysis of NiSA PCNF by X‐Ray photoelectron spectroscopy (XPS) suggests the successful incorporation of N and Ni elements (Figure S6, Supporting Information), where the Ni atom is likely to be in an unsaturated low‐valent state.^[^ ^25^ ^]^

The chemical states and coordination environments of metals were systematically probed *via* the XANES and EXAFS toolboxes. As for the NiSA PCNF, the absorption edge position and white line intensity is located between that of Ni foil and NiO (Figure 1f), implying that the valence state of isolated Ni atoms is between metallic (Ni^0^) and oxidized (Ni^2+^) status. Moreover, in the Fourier‐transformed k3‐weighted *χ*(k) function of the EXAFS (FT‐EXAFS) spectra (Figure 1g), the dominant peak of NiSA PCNF at 1.38 Å corresponds to the Ni–N bonding rather than the Ni–Ni bonding at 2.11 Å or the Ni–O bonding at 1.65 Å. The related fitting results suggest that the Ni center is coordinated with four N atoms (Ni–N_4_) (Figure S7 and Table S2, Supporting Information). In addition, the absorption edge position and white line intensity of CoSA PCNF and FeSA PCNF are also located between those of Co foil/Fe foil and CoO/Fe_2_O_3_, indicating that the valence state of isolated Co/Fe atoms is between metallic (Co^0^)/(Fe^0^) and oxidized (Co^2+^)/(Fe^2+,3+^) status. Similar observations can be found in the FT‐EXAFS spectra of CoSA PCNF and FeSA PCNF with the minor peaks located at 1.32 and 1.53 Å, respectively, which are shifted to the lower *R* direction in comparison with the Co–Co (2.18 Å) and Fe–Fe (2.20 Å) bonding of bulk Fe and Co (Figure S8, Supporting Information). The combination of HAADF‐STEM, EXAFS, and XANES analyses upon MSA PCNF has unambiguously determined the incorporation of NiN_4_C_4_, CoN_4_C_4,_ and FeN_4_C_4_ moieties in fibrous carbon skeletons.

**Figure 2:**
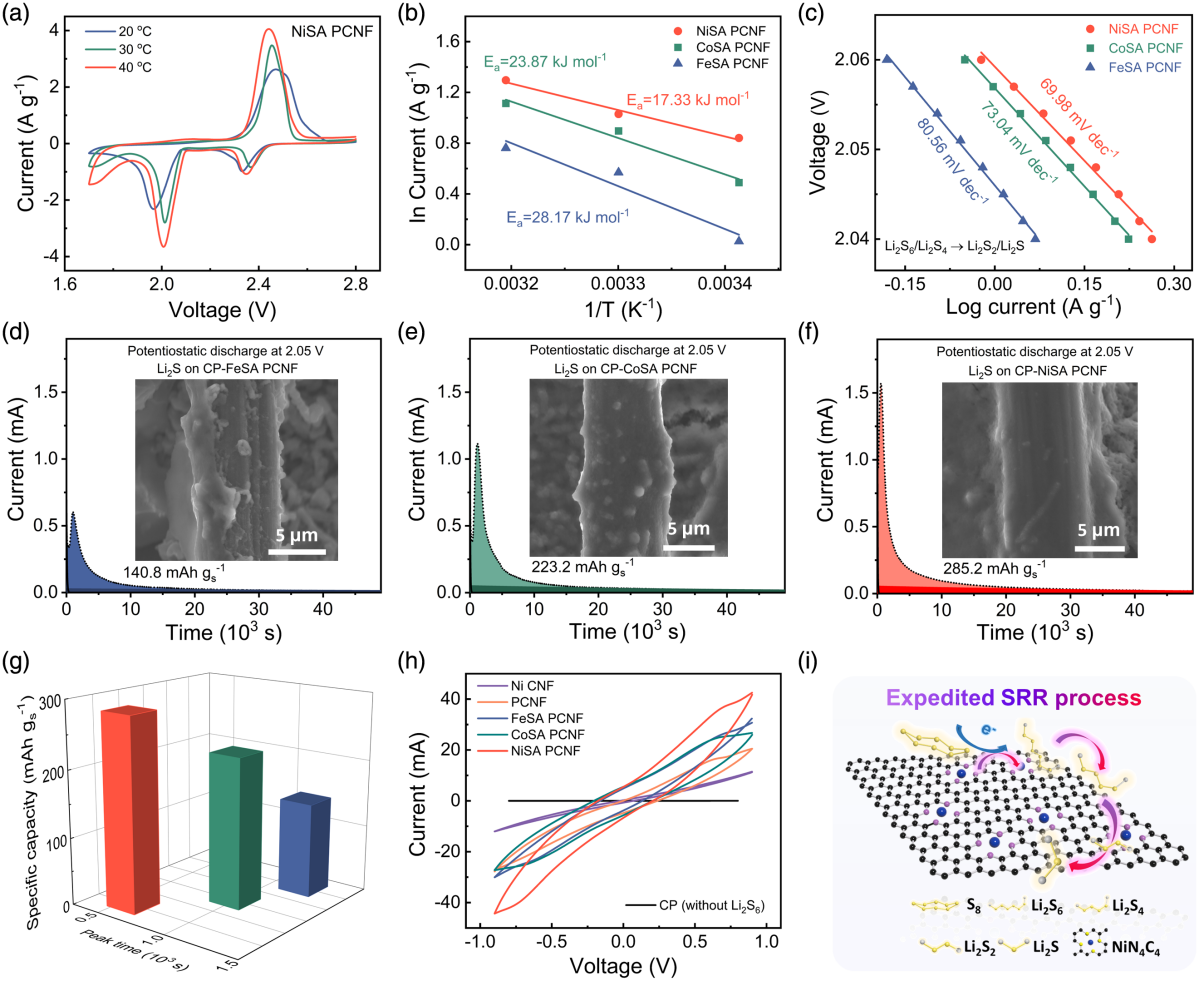
Electrochemical kinetic measurements. a) Temperature‐variable cyclic voltammetry (CV) profiles of NiSA PCNF‐derived cathodes. b) Related plot of Li_2_S_4_ conversion rates as a function of temperatures. c) Tafel plots for the CV peaks between 1.98 and 2.05 V at 30 °C. d–f) Potentiostatic discharge profiles indicating Li_2_S precipitation of d) FeSA PCNF, e) CoSA PCNF, and f) NiSA PCNF at 2.05 V. Inset: Corresponding scanning electron microscopy (SEM) images. g) Specific capacity and peak time of FeSA PCNF, CoSA PCNF, and NiSA PCNF regarding Li_2_S nucleation. h) CV curves of symmetric cells using NiSA PCNF, CoSA PCNF, FeSA PCNF, PCNF, and Ni CNF electrodes in electrolytes with and without Li_2_S_6_ at 50 mV s^−1^. i) Schematic illustration of expedited sulfur reduction reaction (SRR) process catalyzed by NiN_4_C_4_.

To investigate the catalytic conversion efficiency toward the rate‐determining step of SRR, the activation energies of Li_2_S_4_ → Li_2_S conversion reaction were evaluated for MSA PCNF samples. **Figure** **2**a and S9, Supporting Information manifest cyclic voltammetry (CV) profiles of the thus‐derived sulfur cathodes collected at different temperatures (20, 30, and 40 °C). The two featured cathodic peaks appear at 2.3–2.4 and 2.0–2.1 V, which correspond to the generation of soluble LiPSs and insoluble Li_2_S_2_/Li_2_S, respectively. It is evident that NiSA PCNF‐derived cathode possesses a higher redox current in comparison with those of Fe(Co)SA PCNF‐based counterparts, suggesting superior catalytic performance. Considering that the Li_2_S_4_ → Li_2_S conversion process contributes *≈*75% of the theoretical capacity, the activation energy was calculated by the relevant peak appearing at 2.0–2.1 V. The intensity of peak current (*j*) as a function of temperature can be fitted by using the Arrhenius equation:$j\propto A\times e^{-E_{a}/RT}$. In this equation, *E*_a_ is the activation energy, *R* is the gas constant, *A* is a pre‐exponential factor, and *T* is the temperature. The slope of the curve plotted in Figure 2b represents the activation energy for the Li_2_S_4_ → Li_2_S conversion reaction. NiSA PCNF electrode exhibits the lowest activation energy quantity (17.33 kJ mol^−1^), indicating advantageous catalytic capability. Likewise, the NiSA PCNF electrode presents a smaller Tafel slope value of 69.98 mV dec^−1^ as compared to CoSA PCNF (73.04 mV dec^−1^) and FeSA PCNF electrodes (80.56 mV dec^−1^), indicating faster reaction kinetics of Li_2_S_4_ → Li_2_S process (Figure 2c). In further contexts, Li‐ion diffusivity of NiSA PCNF‐, PCNF‐, and Ni CNF‐based sulfur electrodes was evaluated by CV tests with variable scan rates. As depicted in Figure S10, Supporting Information, NiSA PCNF electrode presents a faster Li‐ion diffusion rate in contrast to its counterparts.^[^ ^26^ ^]^

The precipitation of solid Li_2_S product on carbon paper (CP)‐MSA PCNF was further examined via chronoamperometric nucleation test. CP‐NiSA PCNF harvests the highest Li_2_S nucleation capacity of 285.2 mAh g_s_^−1^ and reaches the position of the peak current in the shortest duration, implying prompted SRR reaction kinetics (Figure 2d–f and S11, Supporting Information). To inspect the morphology of Li_2_S deposition, post‐mortem scanning electron microscope (SEM) observations show that the CP‐NiSA PCNF surface is fully covered by Li_2_S precipitation, whereas the CP‐Fe(Co)SA PCNF presents a relatively low Li_2_S coverage (Figure 2d–f inset).^[^ ^27^, ^28^, ^29^, ^30^ ^]^ Encouragingly, NiSA PCNF not only induces the largest Li_2_S nucleation capacity but also enables the earliest peak time, indicative of the most favorable liquid–solid conversion kinetics during the SRR process (Figure 2g).

To comprehensively explore the catalytic ability of MSA catalyst with respect to the full SRR process, CV profiles of MSA PCNF, PCNF, and Ni CNF symmetric cells were collected in the presence/absence of Li_2_S_6_ electrolyte at a scan rate of 0.5 mV s^−1^ (Figure S12, Supporting Information). Obviously, NiSA PCNF enables an advanced electrochemical activity among the tested materials.^[^ ^31^, ^32^, ^33^ ^]^ CV measurements of such symmetric cells at 50 mV s^−1^ between −1.0 and 1.0 V were also carried out (Figure 2h), again confirming the superior catalytic performance of NiSA PCNF.^[^ ^34^ ^]^ The schematic illustration in Figure 2i demonstrates the expedited SRR process catalyzed by NiN_4_C_4_ sites, in which polysulfide conversion and Li_2_S precipitation are greatly accelerated on the NiSA PCNF.

**Figure 3:**
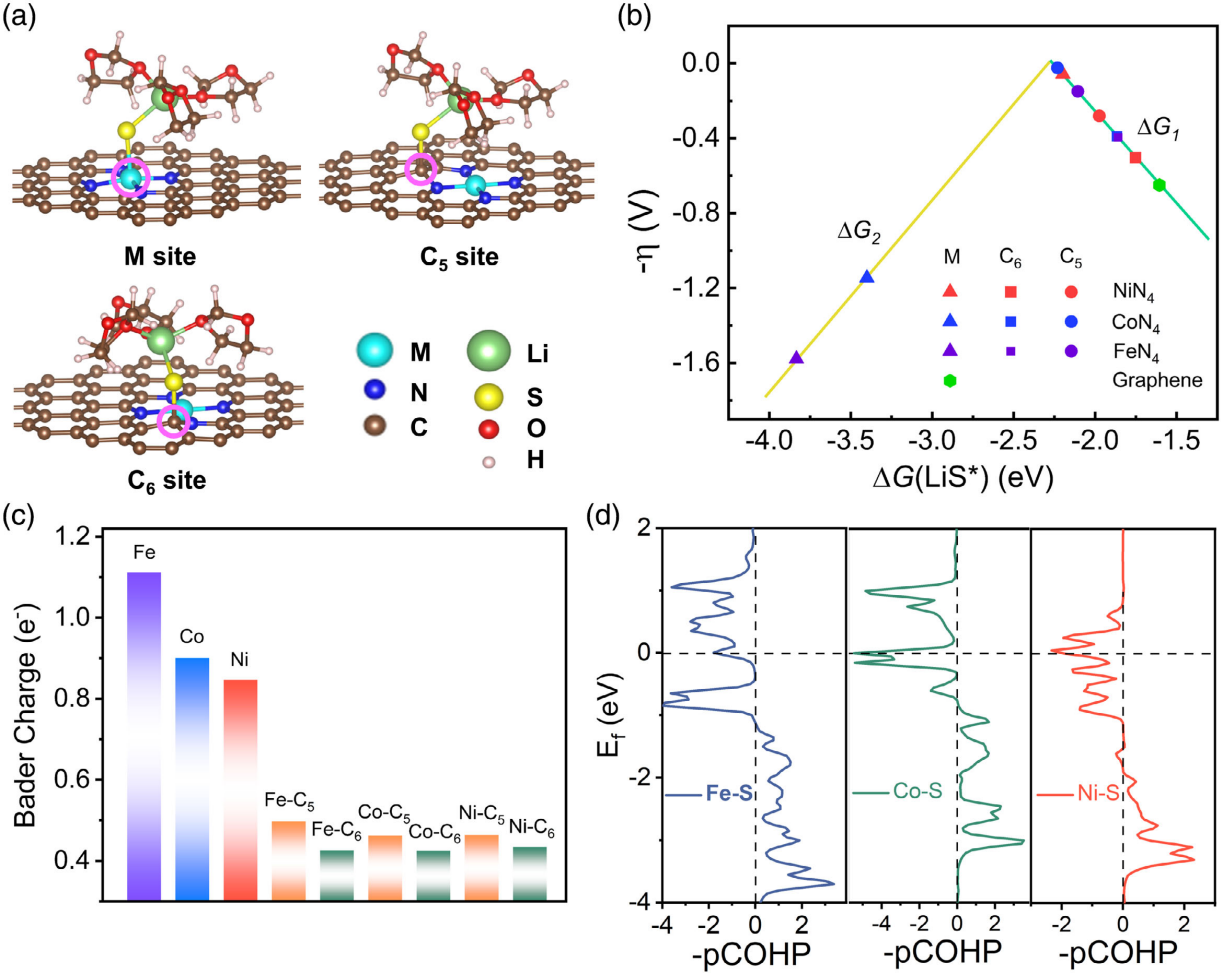
Theoretical simulations of solid–solid reaction process (Li_2_S_2_ + 2Li^+^ + 2e^−^ → 2Li_2_S) on MN_4_‐graphene. a) Optimized adsorption configurations of solvated LiS* intermediate with the first explicit solvation shell (DOL molecule) on three representative sites, e.g., one metal site and two carbon sites in the five‐membered (C_5_) and six‐membered ring (C_6_). b) Volcano curve between the negative overpotential and adsorption free energy of LiS* on the above active sites (triangles, squares, circles, and hexagon indicates the M, C_5_, C_6_ site of MN_4_C_4_ and C site of pristine graphene, respectively). c) Bader charge analysis of M, C_5,_ and C_6_ site in the MN_4_‐graphene. d) Chemical‐bonding analysis of *d* orbitals belonging to the metal site of MN_4_C_4_ and *p* orbitals of S atom from LiS* using pCOHP method. Horizontal dashed line indicates the Fermi level.

SRR process undergoes a complicated multiple‐step evolution. Note that the liquid–solid conversion from Li_2_S_4_ to Li_2_S is extremely sluggish and recognized as the rate‐determining step of the SRR process.^[^ ^35^, ^36^, ^37^, ^38^ ^]^ In this sense, the rational design of electrocatalysts to accelerate the Li_2_S_4_ → Li_2_S conversion is an effective maneuver to maximize sulfur utilization. It is generally received that moderate adsorption (neither too strong nor too weak) of LiPSs on the catalytic sites is a key prerequisite for an efficient catalysis in sulfur electrochemistry.^[^ ^28^ ^]^ This would guide the theoretical simulations to predict the catalytic activity and reaction pathways of SRR process based on the analysis of metal centers and surrounding N/C atoms in M–N–C moieties. To ulteriorly gain insight into the SRR catalytic activity of representative MN_4_C_4_ (M = Fe, Co, or Ni), DFT calculation was employed and MN_4_‐incorporated graphene configurations were constructed beforehand (Figure S13, Supporting Information). Considering that the final solid–solid reaction is the slowest step, such a process (Li_2_S_2_ + 2Li^+^ + 2e^−^ → 2Li_2_S) was accordingly simulated with the aim to examine the catalytic properties of different adsorption sites. Along this line, a LiS* radical intermediate solvated by 1,3‐dioxolane (DOL) molecule was modeled by considering the factually chemical environments (Figure S14, Supporting Information).^[^ ^39^ ^]^ We then considered four possible adsorption sites, namely the metal atom (M) site, the nitrogen atom (N) site, and two carbon atom sites in the five‐membered ring (C_5_)/six‐membered ring (C_6_) adjacent to the N atom.^[^ ^40^ ^]^ It is noted that the LiS* radical shuttles from N site to the M site after relaxation, in contrast to the scenarios at C_5_ and C_6_ sites. **Figure** **3**a depicts optimized adsorption configurations of LiS* intermediate, with the corresponding adsorption Gibbs free energy values (Δ*G*_ads_) summarized in Table S3, Supporting Information. In comparison with a bare graphitic framework, the implant of heterogeneous MN_4_ groups apparently improves the radical anchoring on M–N–C moiety throughout interactions between M/C_5_/C_6_ sites and S atoms. In terms of a fixed metal type, the trend of the adsorption strength for different sites follows: M > C_5_ > C_6_; As for MN_4_‐incorporated graphene based on distinct metal single‐atoms, the binding strength of LiS* follows the order of Fe > Co > Ni.

We then introduced a descriptor of thermodynamic overpotential (*η*) to qualitatively evaluate the catalytic activity of diverse adsorption sites in the two‐step Li_2_S_2_ → Li_2_S conversion reaction (Step 1: 3DOL + Li_2_S_2_ + Li^+^ + e^−^ + * → 3DOL −LiS* + Li_2_S; Step 2: 3DOL − LiS* + Li^+^ + e^−^ + * → 3DOL +Li_2_S + *). Figure 3b displays the relationship between the negative overpotential and free energy of adsorbed LiS* intermediate, which appears as a volcano plot. On the left side of the volcano profile, the determination of potential is limited by reaction Step 2 due to the more negative Δ*G*_ads_ at Fe (−3.83 eV) and Co (−3.40 eV) sites. On the right side, the potential is otherwise determined by reaction Step 1. It is known that the peak position of the volcano profile represents the site affording optimal catalytic activity. As shown in the plot, MN_4_‐graphene falling in the proximity of the optimal of the plot follows the order of C_5_ site in CoN_4_‐graphene, Ni site in NiN_4_‐graphene, and C_5_ site in FeN_4_‐graphene according to the minimized overpotential by 0.02, 0.06, and 0.15 V, respectively. Even though the C_5_ site of CoN_4_‐graphene possesses a lower overpotential value than the Ni site in NiN_4_‐graphene by 0.04 V, the |Δ*G*_ads_| of LiS* on the Co site is obviously higher than that on the C_5_ site by 1.17 eV. In this sense, it would be preferable for the solvated LiS* radical to occupy the Co site rather than the C_5_ site on the CoN_4_‐graphene, suggestive of a single‐site catalytic mechanism, and likewise, the FeN_4_‐graphene. In contrast, the Δ*G*_ads_ of LiS* over the Ni site shows a slight disparity from that over the C_5_ and C_6_ sites, thereby implying a multi‐site catalytic pathway for NiN_4_‐graphene. Collectively, our theoretical prediction indicates that NiSA PCNF harvests superior catalytic activity in terms of boosting Li_2_S_2_ → Li_2_S conversion to the CoSA PCNF and FeSA PCNF counterparts.

To further unveil the origin of high SRR catalytic activity, we modeled the atomic charge distribution at the MN_4_‐graphene framework according to the Bader scheme. As presented in Figure 3c, all the M, C_5_, and C_6_ sites possess positive charges, with the values ranked in descending order of M > C_5_ > C_6_ owing to the larger electronegativity of heteroatom N compared with metal atom and C atom. Considering the polar bonding configuration of LiS* radical with S atom owning negative charges, MN_4_‐graphene carrying a more positive charge would induce the formation of stronger binding of LiS* because of higher Coulomb interactions, which is consistent with the calculation results of adsorption energy. This signifies that atomic charge redistribution by the introduction of a heterogeneous MN_4_ group can enhance the anchoring effect of LiS*. The projected density of states (pDOS) of MN_4_‐graphene was additionally calculated (Figure S15 and S16, Supporting Information). In comparison with pristine graphene, there exists a high population of density of states around the Fermi level in MN_4_‐graphene, which are conducive to augmenting the electrical conductivity. Supplementary spin‐charge density simulations also reveal that Fe(Co)N_4_‐graphene possesses magnetism, mainly contributed by the *d*_z2_ orbital of Fe atom and *d*_*xz*_ orbital of Co atom with the magnetic moment of 1.93 and 0.82 μB, respectively (Figure S17, Supporting Information). The occurrence of magnetism might also help promote the capture of LiS* intermediates.

According to classical bonding theory, the binding strength of LiS* over the metal site is related to the energy level differences between *p* orbitals of S atom in LiS* and *d* orbitals of metal atom in MN_4_‐graphene. The higher number of *d*‐electrons (*N*_d_) for metals, i.e., Fe (*N*_d_ = 6), Co (*N*_d_ = 7), and Ni (*N*_d_ = 8), would trigger lower *d*‐band center locations of the active site. In response, the anti‐bonding states harvest lower energy and higher occupancy, resulting in weaker interactions between adsorbed LiS* and metal adsorption site, and vice versa. Quantitative chemical‐bonding analysis was further carried out by pDOS and projected crystal orbital Hamilton population (pCOHP) method for the S atom of LiS* and adjacent metal atom (Figure 3d and S18, Supporting Information). The integrated pCOHP values up to the Fermi level for Fe**–**S, Co**–**S and Ni**–**S are −1.24 and −1.99 eV, −1.13 and −1.13 eV, −0.54, and −0.73 eV with spin‐up and spin‐down state, respectively, suggesting that the sequence of bonding strength between S atom and metal atom follows Fe–S > Co–S > Ni–S. Along this line, NiN_4_‐graphene affords moderate binding strength with LiS*, which is ultimately helpful to promote SRR catalytic activity. Taken together, theoretical insight was gained into the identification of active sites of single‐atomic M–N–C moieties with respect to catalyzing rate‐determining step of the SRR process, especially the decisive solid–solid conversion.

**Figure 4:**
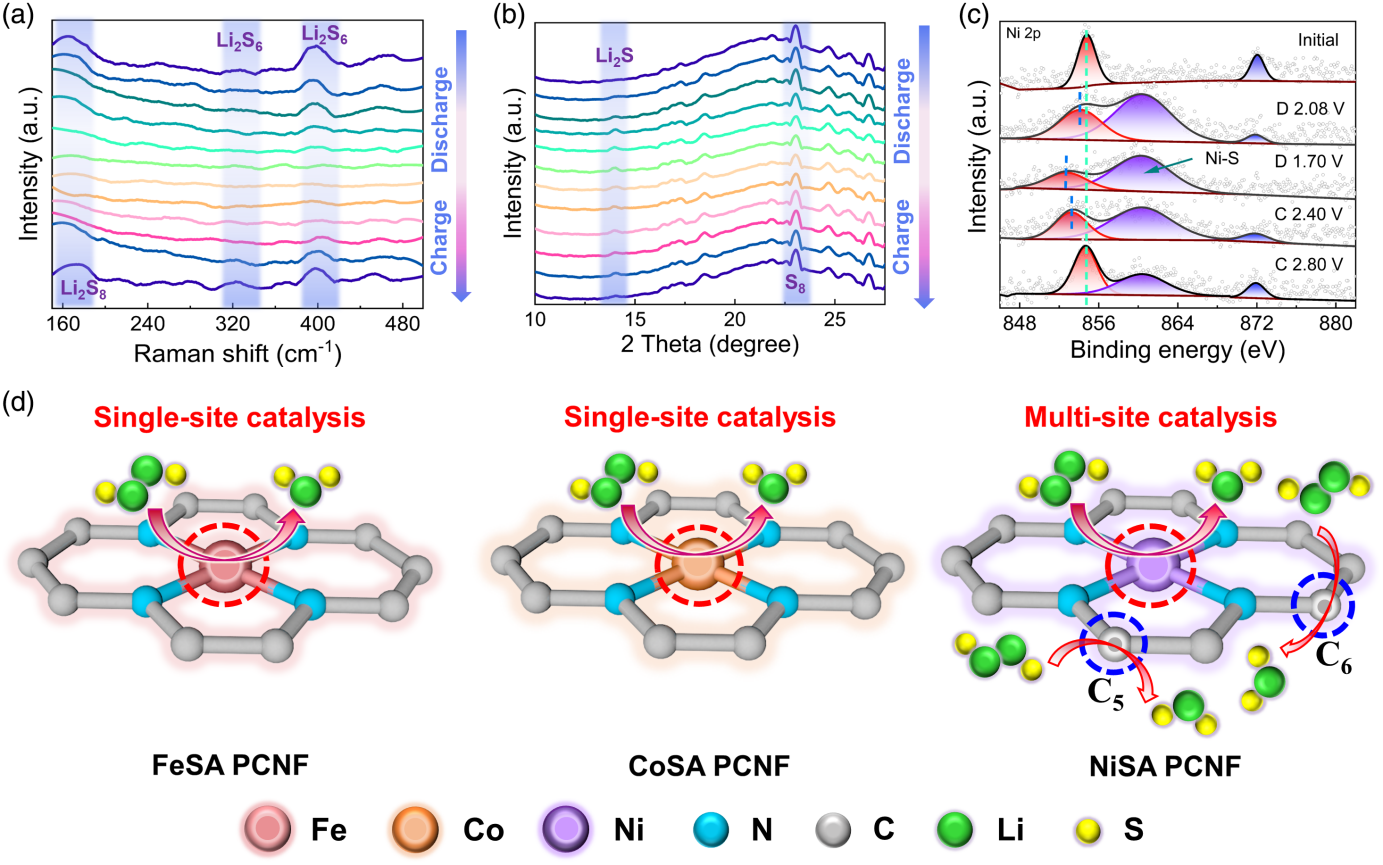
Operando instrumental probing. a) Operando Raman spectra of the electrolyte region at the cathodic side with respect to S/NiSA PCNF during the first discharge/charge cycle. b) In situ XRD patterns of the cathode with respect to S/NiSA PCNF during the first discharge/charge cycle. c) Ex situ XPS Ni 2*p* spectra of the S/NiSA PCNF electrode at selected charge/discharge states. d) Proposed catalytic mechanism toward solid–solid (Li_2_S_2_–Li_2_S) conversion of FeSA PCNF, CoSA PCNF, and NiSA PCNF.

We continued our path to probe the mechanistic features of SRR reaction involving the NiSA PCNF by conducting on‐site instrumental characterizations. The operando Raman spectroscopy and in situ XRD analysis were employed to detect the phase evolution of sulfur species upon discharge/charge. In general, long‐chain LiPSs can be monitored by Raman spectroscopy, where a Li–S cell with a transparent window was assembled to allow in situ detection (Figure S19, Supporting Information). As for our S/NiSA PCNF cathode, Raman intensities of Li_2_S_8_ and Li_2_S_6_ peaks gradually decline in the discharge process and regenerate during charging (**Figure** **4**a), suggestive of good reversibility of sulfur electrochemistry.^[^ ^41^, ^42^ ^]^ This is in stark contrast to the scenario of the control S/Ni CNF cathode, where related Raman signals do not show the conspicuous change (Figure S20, Supporting Information). In situ XRD measurement was further carried out to witness the efficient sulfur redox conversion during the discharge/charge. As shown in Figure 4b, an obvious diffraction pattern at ≈13.6° appears at the end of the discharge process, which can be identified as the formation of Li_2_S. During charging, the intensity of the Li_2_S signal gradually declines and finally disappears.^[^ ^43^, ^44^ ^]^ We performed ex situ XPS studies to further examine any change in surface chemistry of NiSA PCNF electrocatalyst during the first charge/discharge cycle. Figure 4c manifests the ex situ Ni 2*p* spectra of NiSA PCNF. The spectrum at the initial stage shows two characteristic peaks at 854.7 and 872.1 eV, corresponding to the Ni 2*p*_1/2_ and 2*p*_3/2_ of a single Ni atom, respectively. Note that the binding energy of Ni downshifts with the increasing depth of the discharge process and reversibly shifts back upon charging, implying the chemical stability of Ni single atoms during the sulfur redox reaction. When discharging to 2.08 V, a new peak at 862.0 eV in the spectrum is detected, which can be attributed to the formation of Ni–S bonding.^[^ ^45^ ^]^ Collectively, the potential catalytic mechanisms of liquid–solid conversion via the introduction of MN_4_C_4_ moiety are sketched in Figure 4d. We propose whether the C atom sites participate in catalyzing the rate‐determining step of SRR is associated with *N*_d_. As for Fe (*N*_d_ = 6) and Co (*N*_d_ = 7), the Li_2_S precipitation is catalyzed throughout a single‐site mechanism at the sole M site. In contrast, with respect to Ni (*N*_d_ = 8), it is likely that multi‐site (M, C_5_ and C_6_ sites) catalysis occurs for the conversion of Li_2_S_4_ to Li_2_S, thereby maximizing sulfur utilization. This represents a solid example of unambiguous identification in the active sites of MN_4_C_4_‐based M–N–C catalysts harnessing varied electrocatalytic activity toward SRR.

**Figure 5:**
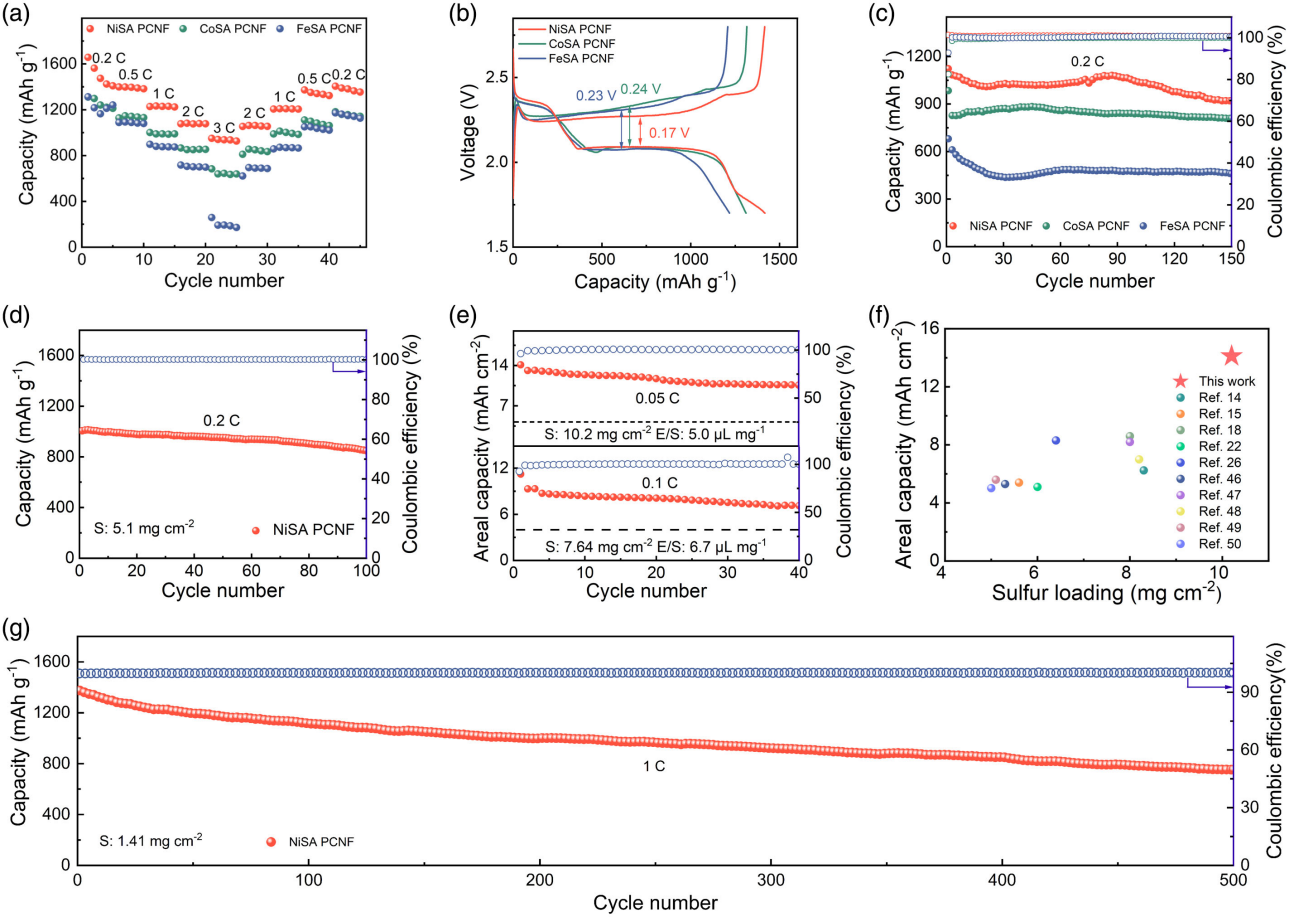
Electrochemical performances of thus‐assembled sulfur electrodes. a) Rate performances of S/NiSA PCNF, S/CoSA PCNF, and S/FeSA PCNF cathodes. b) Galvanostatic charge/discharge (GCD) curves of NiSA PCNF‐, CoSA PCNF‐, and FeSA PCNF‐based cathodes at 0.2 C. c) Cyclic performances S/NiSA PCNF, S/CoSA PCNF, and S/FeSA PCNF cathodes at 0.2 C. d) Cyclic performance of S/NiSA PCNF cathode with a sulfur loading of 5.1 mg cm^−2^ at 0.2 C. e) Cyclic performances of S/NiSA PCNF cathodes with a sulfur loading of 10.20 mg cm^−2^ at 0.05 C and 7.64 mg cm^−2^ at 0.1 C, respectively. f) Comparison of areal capacities of high‐loading cathodes between this work and other reported sulfur cathodes based on SACs. g) Long cycling performance S/NiSA PCNF cathode at 1 C.

We next proceeded to validate the SRR activities of MSA PCNFs by evaluating the electrochemical performances of derived cathodes. As for the three types of S/MSA PCNF electrodes, **Figure** **5**a manifests their rate performances under a typical sulfur loading of 1.8 mg cm^−2^. At varied current densities of 0.2, 0.5, 1.0, 2.0 and 3.0 C, the discharge capacities of S/NiSA PCNF cathode reach 1498.6, 1399.0, 1228.1, 1077.5, and 950.1 mAh g^−1^, respectively. These are markedly superior to those of S/FeSA PCNF and S/CoSA PCNF counterparts, indicating improved rate performance of S/NiSA PCNF. Such an outstanding rate capability compares favorably with those of reported SACs‐derived cathodes in the Li–S realm, especially at a high‐rate (e.g., 3.0 C) condition (Table S4, Supporting Information). The catalytic superiority of NiSA PCNF was further verified by the galvanostatic charge/discharge (GCD) test at a constant current density of 0.2 C (1.0 C = 1675 mA g^−1^). As shown in Figure 5b, all MSA PCNF cathodes manifest two discharge plateaus. The valley that appears between the first plateau and the second plateau was defined as the Li_2_S nucleation point. The potential gap between the Li_2_S nucleation point and the tangential line of the potential plateau is used to evaluate the Li_2_S nucleation. Obviously, the NiSA PCNF cathode possesses a low overpotential of 11.8 mV, suggesting a low interfacial energy barrier existed for Li_2_S nucleation and deposition on the surface of NiSA PCNF. The increased Li_2_S formation during the discharging process is further evidenced by the onset potential of charge curves. A lower onset potential and longer active time could be observed for NiSA PCNF cathode, indicating more Li_2_S produced during the discharging process. The voltage gap of the NiSA PCNF cathode remains the lowest value of only 0.17 V, suggesting a kinetically favorable reaction process. Notably, the NiSA PCNF electrode enables the highest capacity at the final solid–solid conversion step, which might originate from the advanced electrocatalytic effect modulated by the multi‐site electrocatalytic mechanism that is proposed aforementioned.

Cyclic stability of the three types of S/MSA PCNF electrodes was also tested. As depicted in Figure 5c, the S/NiSA PCNF electrode manages to deliver an initial capacity of 1122.9 mAh g^−1^ with a low capacity decay of 0.12% per cycle over 150 cycles at 0.2 C, indicative of the advanced ability of NiSA PCNF to restrain the “shuttle effect” as compared to Fe(Co)SA PCNFs and Ni CNF (Figure S21 and S22, Supporting Information). In this sense, our experimental evaluation results of electrochemical performances are in good agreement with theoretical simulations, corroborating the impressive SRR activity of NiSA PCNF.

To envisage practical applications, Li–S cells based on the representative S/NiSA PCNF cathode with elevated sulfur loadings (5.0–10.2 mg cm^−2^) were constructed. When the sulfur loading reaches 5.1 mg cm^−2^, S/NiSA PCNF cathode delivers an initial capacity of 1005.8 mAh g^−1^ and maintains a capacity of 849.1 mAh g^−1^ after 100 cycles at 0.2 C (Figure 5d). Such a cathode also enables an excellent rate capability: when cycled at 0.1, 0.2, 0.5, and 1.0 C, it harvests discharge capacities of 1059.2, 939.1, 869.6, and 509.1 mAh g^−1^, respectively (Figure S23, Supporting Information). As shown in Figure 5e, under an elevated sulfur loading of 10.2 mg cm^−2^ and a lean‐electrolyte condition (E/S ratio ≈ 5.5 μL mg^−1^), S/NiSA PCNF cathode with a thickness of 150 μm achieves a high initial areal capacity of 14.12 mAh cm^−2^ and still maintains a capacity of 10.58 mAh cm^−2^ after 40 cycles at 0.05 C (Figure S24, Supporting Information), readily surpassing the typical benchmark value of commercial Li‐ion battery (≈4 mAh cm^−2^). More impressively, as shown in Figure 5f, this outstanding areal capacity exhibits an overwhelming advantage over the previously reported sulfur cathodes based on SACs.^[^ ^46^, ^47^, ^48^, ^49^, ^50^, ^51^ ^]^ In terms of long cycling performance, S/NiSA PCNF cathode is able to retain a high capacity of 754.4 mAh g^−1^ and achieve a low capacity decay rate of 0.09% per cycle after 500 cycles at 1.0 C, indicative of excellent cyclic stability (Figure 5g). In addition, a 2.0 cm × 3.0 cm pouch cell was further assembled based on a S/NiSA PCNF cathode at a sulfur loading of ≈2.0 mg cm^−2^, upholding the continuous powering of a light emitting diode (LED) indicator under different bending states (Figure S25, Supporting Information).

## 3 Conclusions

In summary, a universal route was developed to synthesize MSA PCNFs (M = Fe, Co, or Ni) as electrocatalytic hosts to steer the rate‐determining step of the SRR process. The coordination configurations of MN_4_C_4_ moiety have been identified by HAADF‐STEM, XANES, and EXAFS, which aid in elucidating the structure of obtained single atoms at the atomic level. Based on exhaustive theoretical simulations and electrokinetic analysis, it is disclosed that NiSA PCNF exhibits a robust electrocatalytic effect on SRR affording a close collaboration of Ni atomic center and adjacent carbon atoms. This unique multi‐site catalytic mechanism is distinct from the single‐site modulation of Fe(Co)SA PCNF, accordingly endowing the NiSA PCNF with excellent electrocatalytic performances toward the liquid–solid redox conversion. The thus‐constructed Li–S cells deliver a high capacity of 950.1 mAh g^−1^ at 3.0 C. This work offers a meaningful roadmap for guiding the rational design of single‐atom catalysts to boost sulfur electrocatalysis, holding promise in the pursuit of practical Li–S systems.

## Conflict of Interest

The authors declare no conflict of interest.

## Supporting information

Supplementary Material

## Data Availability

Research data are not shared.
