# Supplementary material for: Demystifying Activity Origin of M–N–C Single‐Atomic Mediators Toward Expedited Rate‐Determining Step in Li–S Electrochemistry
Source: Small Sci. 2022 Aug 21;2(10):2200059. doi: 10.1002/smsc.202200059 (PMC11935855; doi:10.1002/smsc.202200059)
Supplement: Supplementary file 1 — Supplementary Material [file SMSC-2-2200059-s001.pdf]

Supporting Information

Demystifying Activity Origin of M−N−C Single-Atomic Mediators toward Expedited Rate-Determining Step in Li–S Electrochemistry

Jia Jin, Zhongti Sun, Tianran Yan, Zixiong Shi*, Meiyu Wang, Ting Huang, Yifan Ding, Jingsheng Cai, Peng Wang, Liang Zhang*, and Jingyu Sun**

J. Jin, Z. X. Shi, T. Huang, Y. F. Ding, J. S. Cai, J. Y. Sun

College of Energy, Soochow Institute for Energy and Materials InnovationS (SIEMIS), Light Industry Institute of Electrochemical Power Sources, Jiangsu Provincial Key Laboratory for Advanced Carbon Materials and Wearable Energy Technologies, Soochow University, Suzhou 215006, P. R. China

E-mail: sunjy86@suda.edu.cn

Z. T. Sun

School of Materials Science and Engineering, Jiangsu University, Zhenjiang 212013, P. R. China

E-mail: ztsun@ujs.edu.cn

T. Y. Yan, L. Zhang

Institute of Functional Nano & Soft Materials (FUNSOM), Jiangsu Provincial Key Laboratory for Carbon-Based Functional Materials and Devices, Soochow University, Suzhou 215006, P. R. China

E-mail: liangzhang2019@suda.edu.cn

Z. X. Shi

Materials Science and Engineering, Physical Science and Engineering Division, King Abdullah University of Science and Technology (KAUST), Thuwal, 23955-6900 Saudi Arabia

E-mail: zixiong.shi@kaust.edu.sa

M. Y. Wang, P. Wang

College of Engineering and Applied Sciences and Collaborative Innovation Center of Advanced Microstructures, National Laboratory of Solid-State Microstructures, Jiangsu Key Laboratory of Artificial Functional Materials, Nanjing University, Nanjing 210093, P. R. China

Keywords: single-atomic mediator, M–N–C, sulfur reduction reaction, rate-determining step, Li–S batteries.

Experimental Section

Preparation of ZIF-8.

ZIF-8 crystals were prepared via adding solution A (including 16 mL deionized water and 7.9 mmol $Zn(NO_3)_2·6H_2O$) into solution B (containing 160 mL deionized water and 553 mmol 2-methylimidazole). Upon vigorous stirring for 5 min at room temperature, the product was collected by centrifugation, washed by deionized water over five times, and finally dried in vacuum at 60 °C for 12 h.

Preparation of MSA PCNF, PCNF and Ni CNF.

To synthesize MSA PCNF, 1.5 g polyacrylonitrile, 1.5 g ZIF-8, and 30 mg metal nitrite ($Ni(NO_3)_2·6H_2O$, $Co(NO_3)_2·6H_2O$, or $Fe(NO_3)_3·6H_2O$) were dispersed in 15 mL N,N-dimethylformamide under vigorous stirring. The homogenous mixture was further transferred into a plastic syringe. The electrospinning process was carried out under an applied positive voltage of 16 kV. The as-spun raw fibers were pre-oxidized in air at 260 °C for 2 h and then carbonized at 800 °C for 3 h under Ar atmosphere with a heating rate of 5 °C min^{-1}. The aggregated metallic species were removed in 3.0 M H_2SO_4 solution for 10 h. For comparison, Ni CNF was derived from a similar procedure without the adding of ZIF-8. PCNF

was obtained throughout a similar process except for the adding of metal nitrite. Note that a higher carbonization temperature of 900 °C was employed for preparing PCNF.

Fabrication of sulfur electrodes.

0.25 g sulfur was dissolved in 5 mL CS_2 to form a uniform solution. The as-prepared solution was then evenly dropped onto various fibrous hosts, which were further heated at 155 °C for 12 h in a confined space. Note that the high mass loading cathodes were realized by stacking a pack of fibrous films.

Li_2S nucleation tests.

0.2 mol L^{-1} Li_2S_8 solution was prepared by dissolving Li_2S and sulfur with a molar ratio of 1:7 in a tetraglyme solvent under vigorous stirring at 60 °C for 24 h. NiSA PCNF, CoSA PCNF, FeSA PCNF, Ni CNF, and PCNF were dispersed into ethanol, which were respectively loaded onto carbon paper. As-prepared electrode was employed as the cathode and Li foil used as the anode. 20 μL Li_2S_8 electrolyte and 20 μL LiTFSI (1 mol L^{-1}) were dropped onto the cathode and anode side, respectively. Finally, the assembled cells were galvanostatically discharged to 2.05 V at 0.112 mA and subsequently kept potentiostatically at 2.05 V until the current decreased below 10^{-5} A.

Symmetric cell measurements.

Li_2S_6 symmetric cells were assembled in a glovebox filled with Ar. The as-prepared electrodes were employed as identical electrodes and assembled into a CR2032 coin cell with a Celgard 2400 membrane serving as the separator. 40 μL Li_2S_6 solution (0.6 M) was used as the electrolyte. CV measurements of symmetric cells were performed at a scan rate of 50 mV s^{-1} within the potential range of −1.0 to 1.0 V. Note that CV tests of these symmetric cells at a scan rate of 0.5 mV s^{-1} within the potential range of −1.0 to 1.0 V employed the less concentrated Li_2S_6 solution as the electrolyte (0.2 M).

Electrochemical measurements.

Typical batteries were assembled with S/NiSA PCNF (S/CoSA PCNF, S/FeSA PCNF, S/Ni CNF, or S/PCNF) as the cathode, Li metal as the anode, and Celgard 2400 as the separator. The electrolyte was 0.5 M LiTFSI dissolved in DME/DOL (1:1; V/V) with 0.5 M $LiNO_3$. CV profiles were recorded on the PARSTAT 3000A electrochemical station in a voltage range of 1.7–2.8 V. The galvanostatic charge/discharge profiles, rate and cycling performance results were collected on a LAND CT2001A battery testing system.

Characterizations.

The high-resolution HAADF-STEM images were captured by a TEM (FEI Titan3 G2 60-300) equipped with double spherical aberration correctors. XRD patterns of the composites were obtained on a Bruker D8 Advance diffractometer. XPS spectra were collected by an Escalab 250Xi spectrophotometer, where all binding energy values were calibrated by C 1s = 284.6 eV as the reference. ICP-AES data was collected on an OPTIMA 8000 instrument to analyze the elemental content in synthesized materials. The Fe, Co, and Ni K-edge XAFS spectra were obtained at beamline 11B of Shanghai Synchrotron Radiation Facility and beamline 1W1B of Beijing Synchrotron Radiation Facility. The XAFS spectra were collected in the fluorescence mode at room temperature with the corresponding metal foils as references for energy calibration. The data were processed and fitted using the Ifeffit-based Athena and Artemis programs. The spectra were calibrated, aligned, and normalized with the background removed. The $\chi(R)$ results were modeled using single scattering paths calculated by FEFF6.

Theoretical calculations.

All the first-principles calculations were performed by Vienna Ab-initio Simulation Package (VASP)[1] with projector augmented wave (PAW) pseudopotential[2] and GGA-PBE functional[3]. The dDsC dispersion corrections[4] were introduced to accurately assess the adsorption of LiPSs. Kinetic energy cutoff with spin-polarized plane wave basis set was 500 eV with the ACCURATE precision. To accurately

describe the factual electrochemical surroundings, hybrid solvation model was involved by using the first explicit solvation shell with three DOL molecules, referring to the previous report by Peng et al. and implicit continuum dielectric model as implemented in the VASPsol package[5]. The surface tension parameter was set to be 0 to get rid of electrostatic potential fluctuations in vacuum. Periodical repeating (6×6) graphene supercell was constructed with the vacuum layer thickness of 20 Å to avoid the imaginary interactions along the z direction. All the structure models were fully relaxed until the total energy and residual force per atom was less than 10^{-5} eV and 0.02 eV/Å, respectively. The first-Brillouin zone integrations were sampled by 3 × 3 × 1 grid size for geometric optimization and 5 × 5 × 1 for static computations. Atomic charges were calculated according to the atom-in-molecule scheme proposed by Bader[6]. Chemical bonding analysis were executed by the computer program LOBSTER[7] (Local Orbitals Basis Suite Towards Electronic Structure Reconstruction). In this work, the solid-solid phase transition ($Li_2S_2 \rightarrow Li_2S$) in the SRR process was highlighted. The total reaction can be expressed as:

$Li_2S_2 + 2Li^+ + 2e^- \rightarrow 2Li_2S$

In this sense, two elementary steps are considered:

i) $Li_2S_2 + * + Li^+ + e^- + 3DOL \rightarrow 3DOL\text{-}LiS* + Li_2S$

ii) $3DOL\text{-}LiS* + Li^+ + e^- \rightarrow 3DOL + Li_2S + *$

where the "*" indicates the adsorption site of MN_4-doped graphene, DOL is the major component of electrolyte for Li–S battery, 3DOL-LiS* is the intermediate of solid-solid conversion process. Li_2S_2 and Li_2S are all considered in solid state, where the structure of Li_2S_2 are referred to the previously hypothesized structure[8].

The reaction free energies mentioned above can be calculated in the following:

$\Delta G(LiS*) = G(3DOL\text{-}LiS*) - 3G(DOL) - G(*) - G(Li) - G(Li_2S_2) + G(Li_2S)$

$\Delta G = 2G(Li_2S) - G(Li_2S_2) - 2G(Li)$

$\Delta G_1 = \Delta G(LiS^*)$

$\Delta G_2 = 3G(DOL) + G(Li_2S) + G(^*) - G(3DOL\text{-}LiS^*) - G(Li)$

$\Delta G_2 = \Delta G - \Delta G_1$

where G(Li) stands for the total energy of $Li^+ + e^-$ in the condition of 0 V vs. Li/Li^+ electrode potential. For all the solid-state species, the free energy corrections were also involved using vibrational frequency calculations based on harmonic approximations, G = E + ZPE – TS. The Gibbs free energy of DOL molecule and surface adsorbed species were approximated as energy due to the cancelation before and after reaction. The free energy corrections of adsorbed LiS* intermediates on the different sites including metal site, C atom site on the five-membered ring (C_5) and six-membered ring (C_6) were treated by Vaspkit program[9,10], with 0.03, 0.06 and 0.06 eV, respectively. To qualitatively evaluate the figure of merits of different sites on the MSA PCNF (M = Fe, Co, or Ni), the overpotential (η) was calculated using the formula: $\eta = U_{eq} - \min(\Delta U_i)$, where $U_{eq} = -\Delta G/2e = 2.26$ V; $U_i = -\Delta G_i/e$.

Supporting Figures and Tables

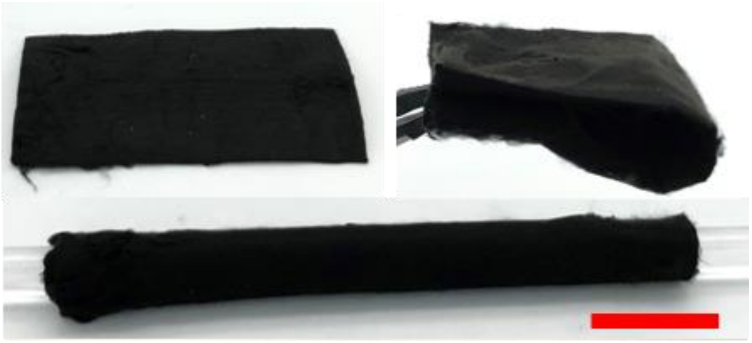

Figure S1. Digital photos of NiSA PCNF. Scar bar: 3 cm.

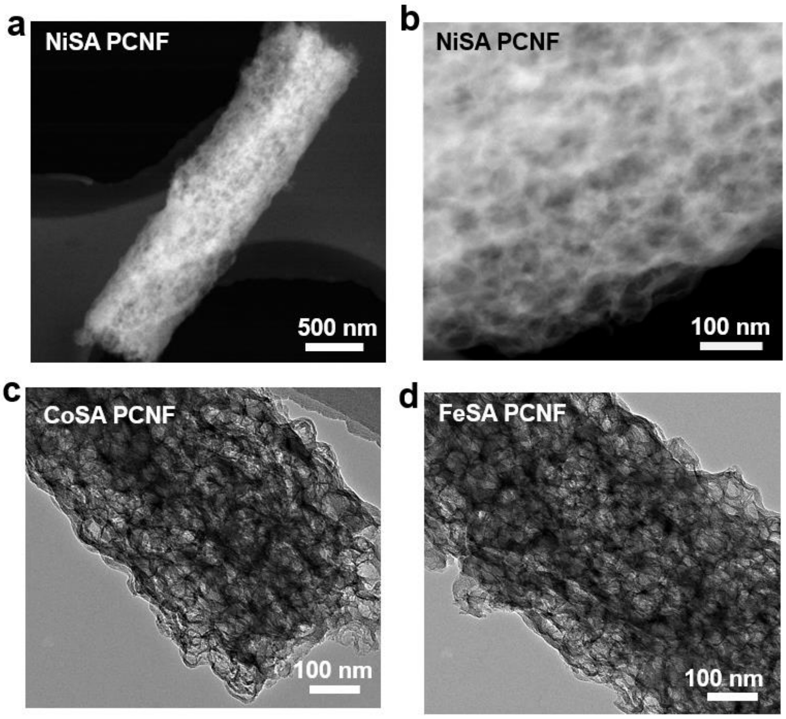

Figure S2. TEM images of a,b) NiSA PCNF, c) CoSA PCNF, and d) FeSA PCNF.

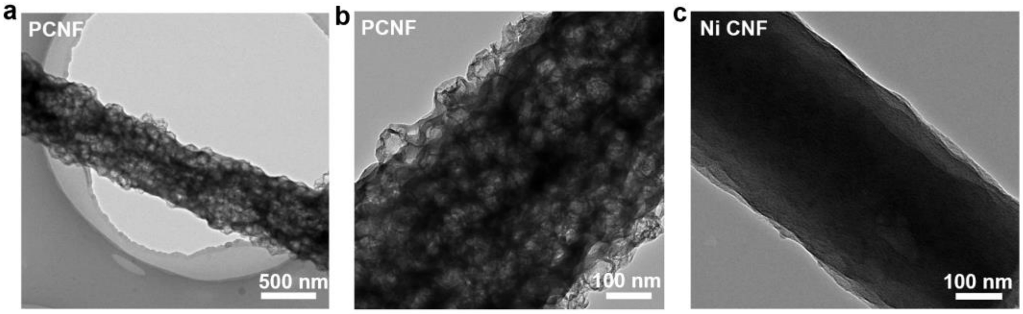

Figure S3. TEM images of a,b) PCNF and c) Ni CNF.

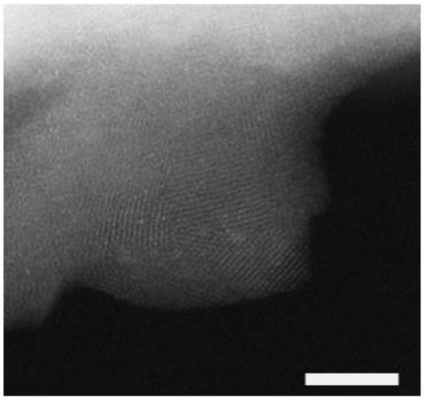

Figure S4. HRTEM image of NiSA PCNF. Scar bar: 3 nm.

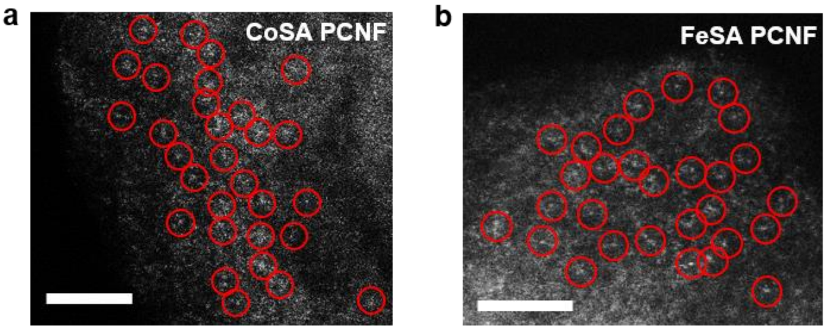

Figure S5. HAADF-STEM images of a) CoSA PCNF and b) FeSA PCNF. Scar bars: 3 nm.

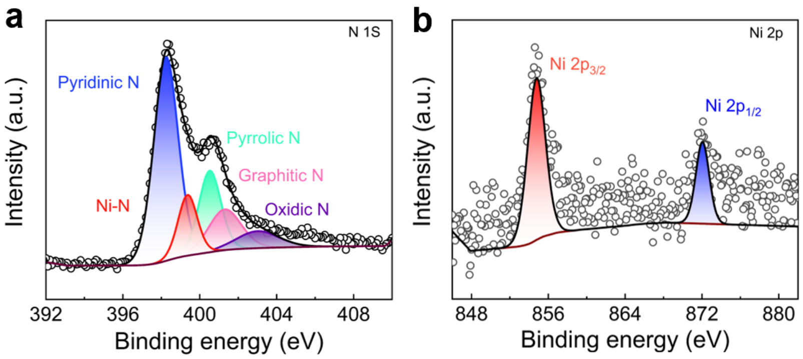

Figure S6. a) N 1s and b) Ni 2p XPS spectra of NiSA NCNF, respectively.

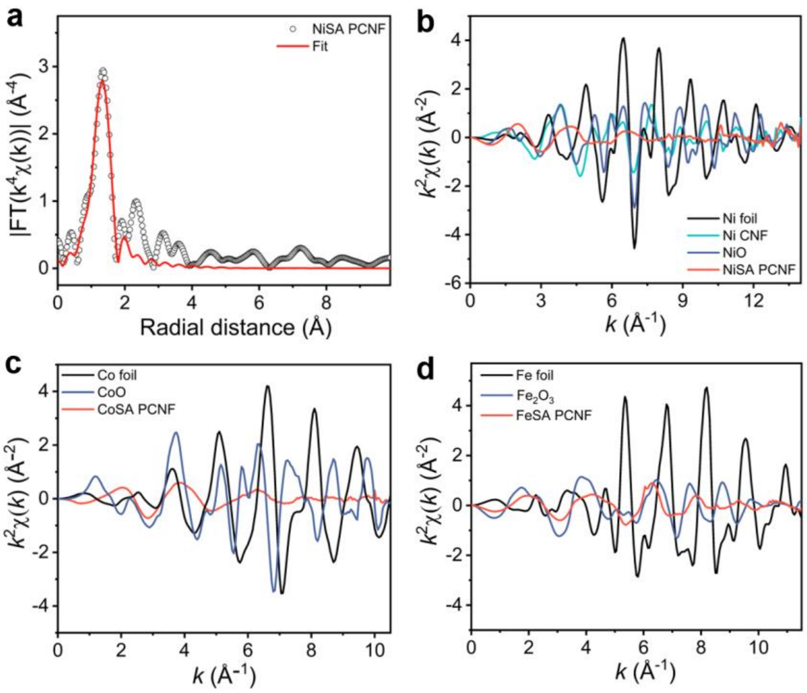

Figure S7. a) Fitting for the EXAFS data of NiSA PCNF. M K-edge EXAFS analysis of MSA PCNF in *k* spaces of b) Ni, c) Co and d) Fe.

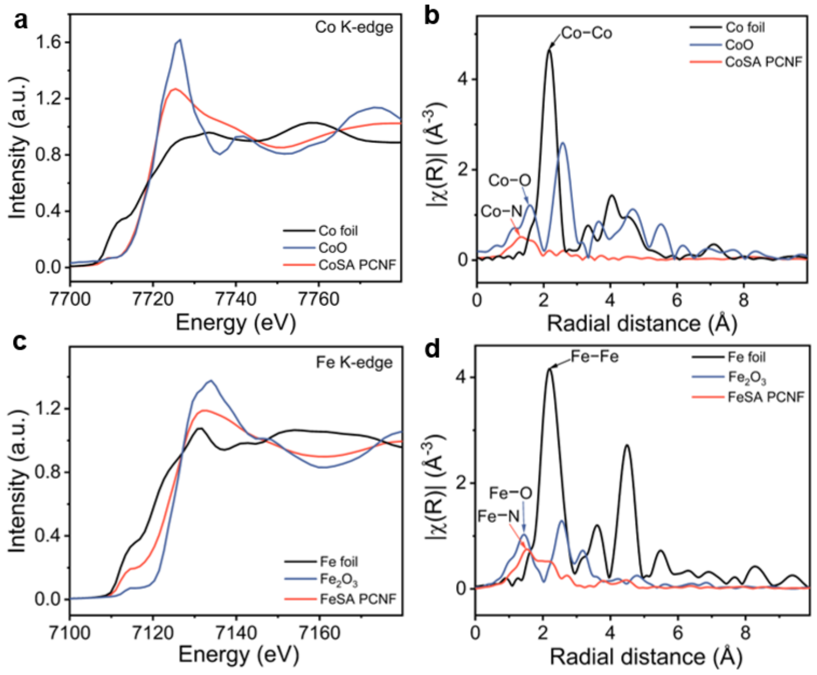

Figure S8. a) XANES for CoSA PCNF, Co foil, and CoO. b) FT-EXAFS in R space for CoSA PCNF, Co foil, and CoO. c) XANES for FeSA PCNF, Co foil, and CoO. d) FT-EXAFS in R space for FeSA PCNF, Fe foil, and FeO.

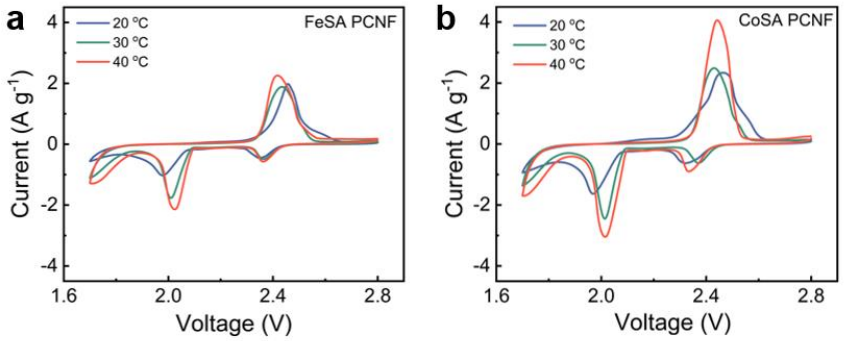

Figure S9. Temperature-variable CV profiles of a) FeSA PCNF- and CoSA PCNF-derived cathodes.

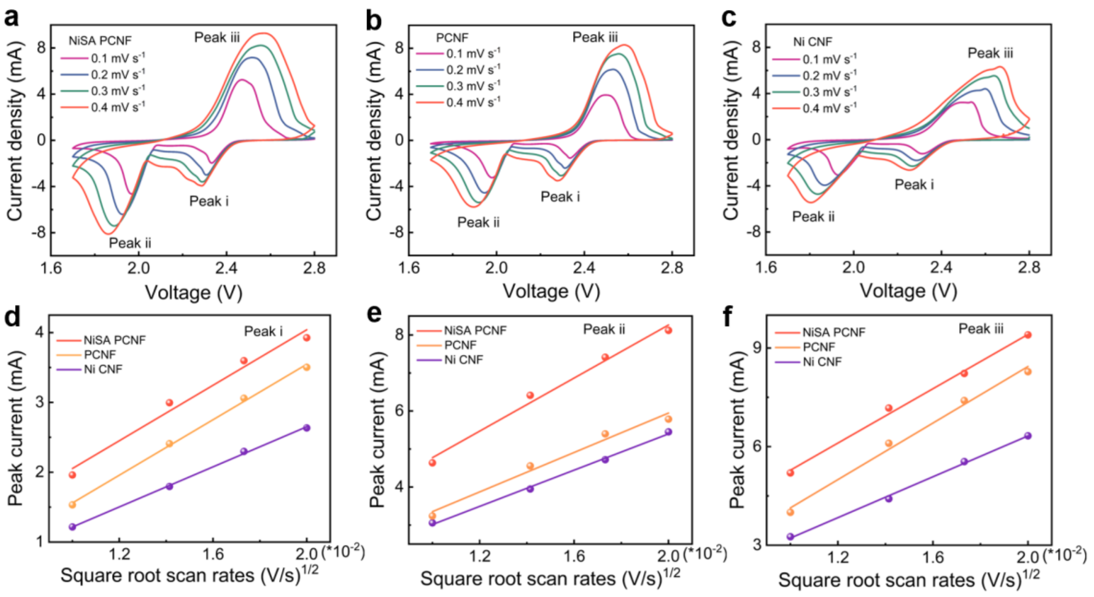

Figure S10. a-c) CV profiles of a) NiSA PCNF b) PCNF, and c) Ni CNF at different scan rates, respectively. d-f) Li-ion diffusion properties of a) peak i, b) peak ii, and c) peak iii, respectively.

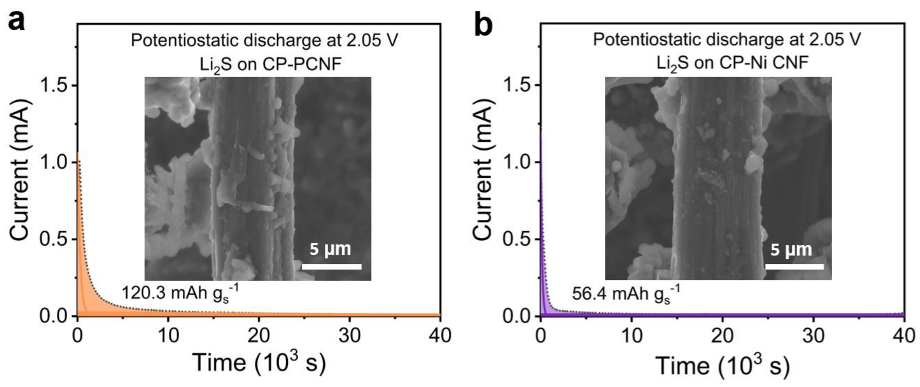

Figure S11. Potentiostatic discharge profiles of a Li_2S_8/tetraglyme solution on a) CP-PCNF and b) CP-Ni CNF surfaces at 2.05 V, respectively. Inset: Corresponding SEM images.

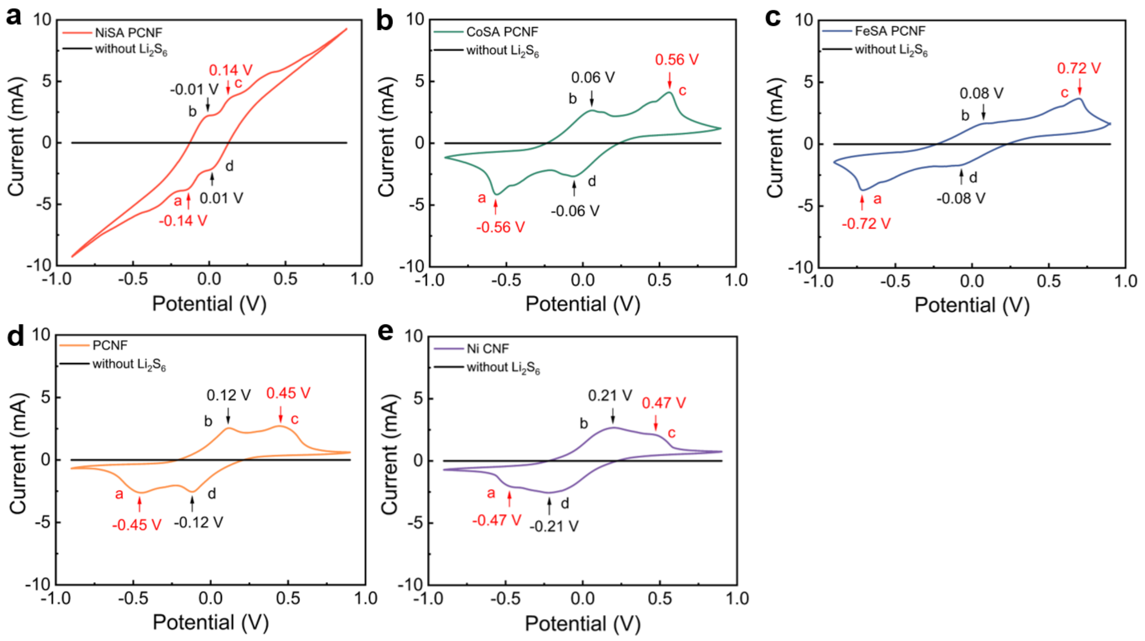

Figure S12. CV profiles of symmetric cells of a) NiSA PCNF, b) CoSA PCNF, c) FeSA PCNF, d) PCNF, and e) Ni CNF in electrolytes with and without 0.2 M Li_2S_6 at 0.5 mV s^{-1}.

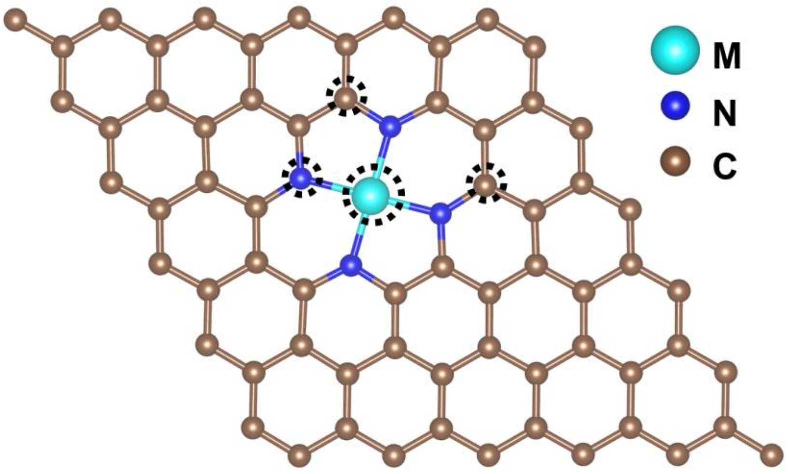

Figure S13. Possible adsorption sites of LiS intermediate on the MN_4C_4 (M = Fe, Co, and Ni). Cyan, blue, and brown ball indicates metal, nitrogen, carbon atom, respectively. Black dashed circle marks possible adsorption sites. There are three possible adsorption sites that are considered: metal (M) site, nitrogen (N)

site, two carbon sites adjacent to nitrogen atoms in the five-membered ring (C_5) and six-membered ring (C_6).

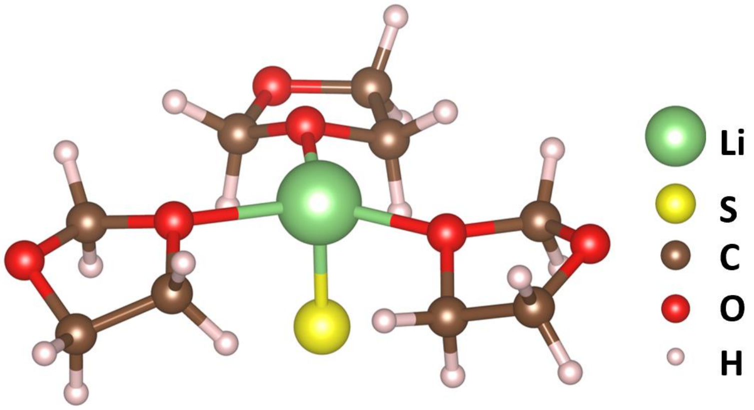

Figure S14. Solvated LiS* configuration with three explicit solvation molecules (DOL).

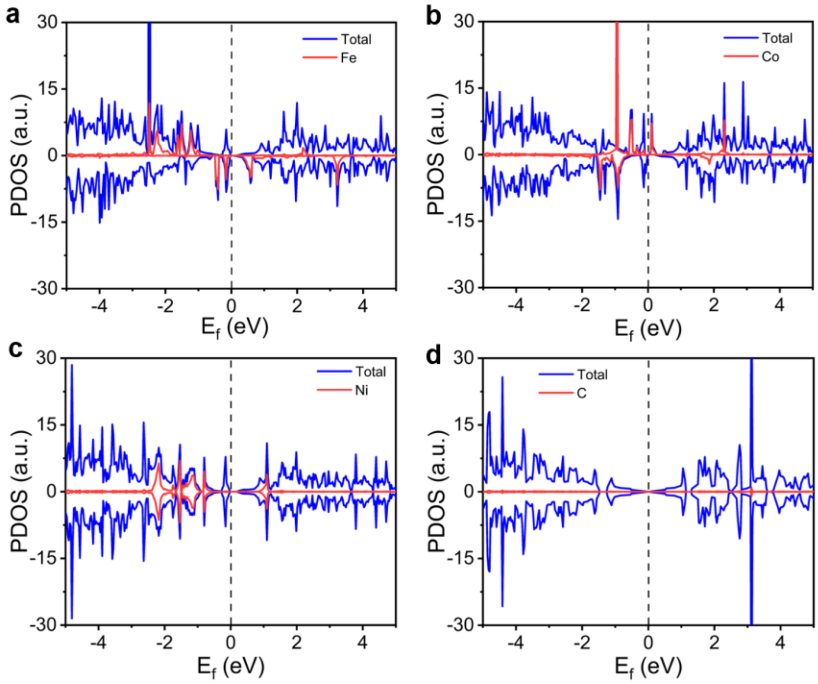

Figure S15. PDOS of total and transition metal for the a) FeN_4C_4, b) CoN_4C_4, c)NiN_4C_4, and d) pristine graphene. Black dashed line marks the Fermi level.

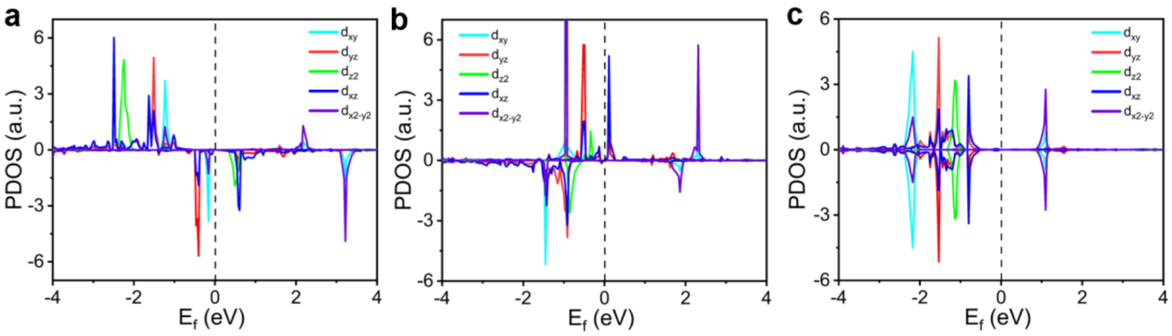

Figure S16. PDOS of a) Fe of FeN_4C_4, b) Co of CoN_4C_4, and c) Ni atom of NiN_4C_4.

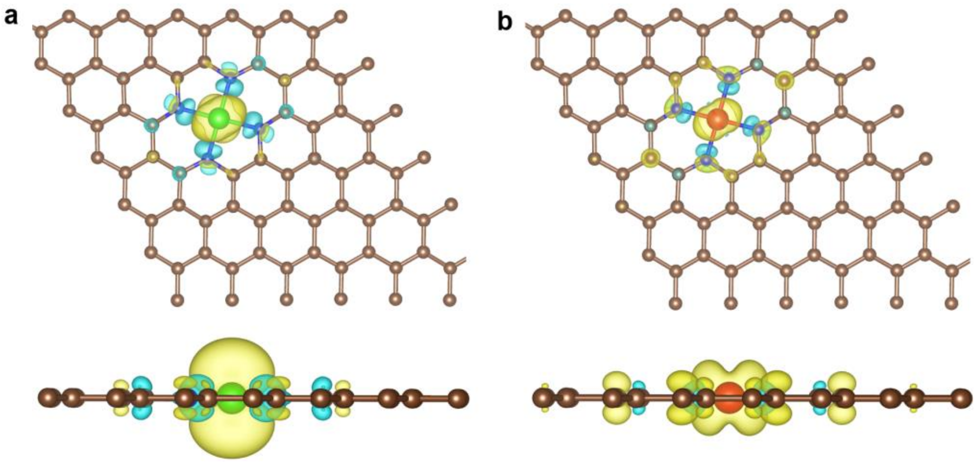

Figure S17. Spin charge density contour of a) FeN_4C_4 and b) CoN_4C_4. The iso-surface value is 0.001 e/bohr^3. Yellow and cyan contour marks spin-up and spin-down state.

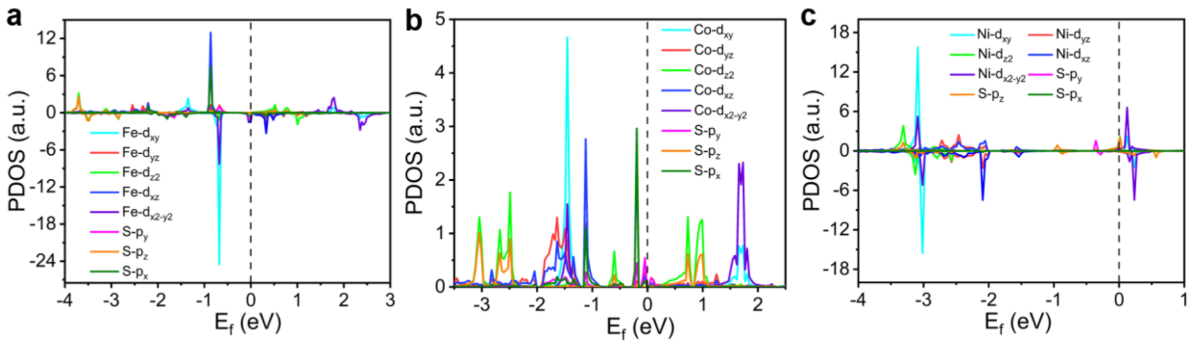

Figure S18. PDOS of *d* orbitals of metal atom and *p* orbitals of S atom on a) FeN_4C_4, b) CoN_4C_4, and c) NiN_4C_4.

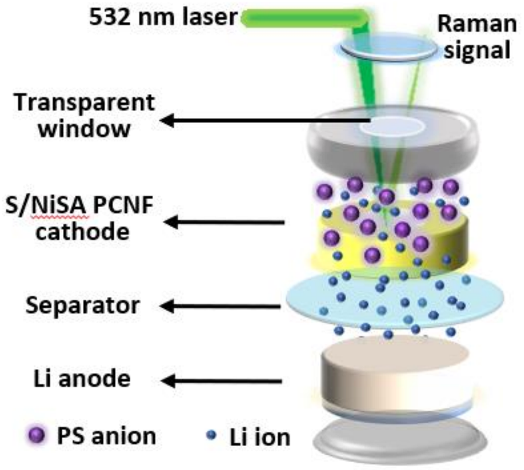

Figure S19. Schematic diagram of the *operando* Raman measurement.

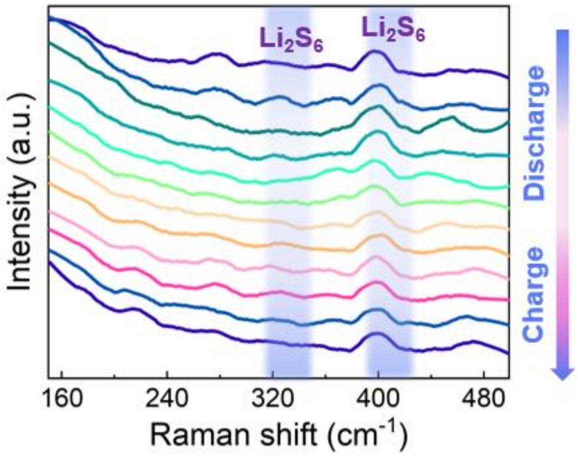

Figure S20. *Operando* Raman spectra of the electrolyte region at the cathodic side with respect to S/Ni CNF during the first discharge/charge cycle.

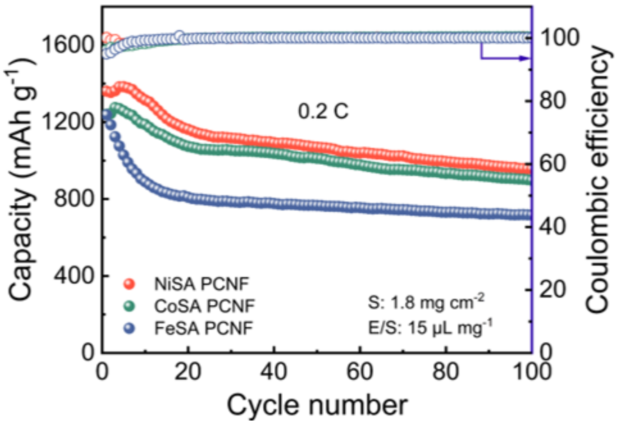

Figure S21. Cycling performances of NiSA PCNF, CoSA PCNF and FeSA PCNF cathodes at 0.2 C.

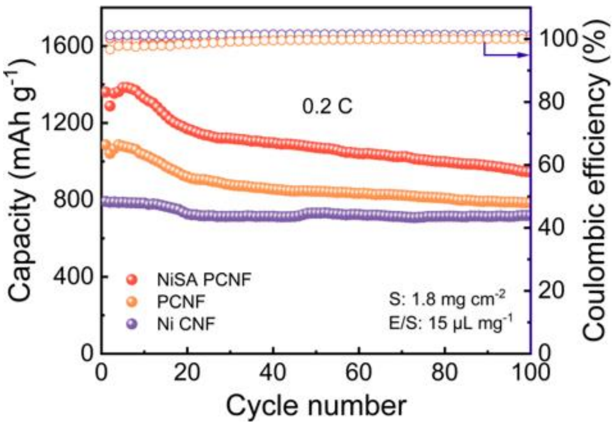

Figure S22. Cycling performances of NiSA PCNF, PCNF, and Ni CNF cathodes at 0.2 C.

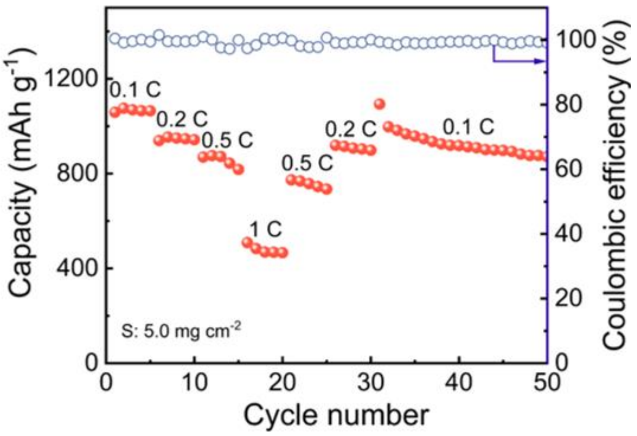

Figure S23. Rate capabilities of NiSA PCNF at a sulfur loading of 5.0 mg cm^{-2}.

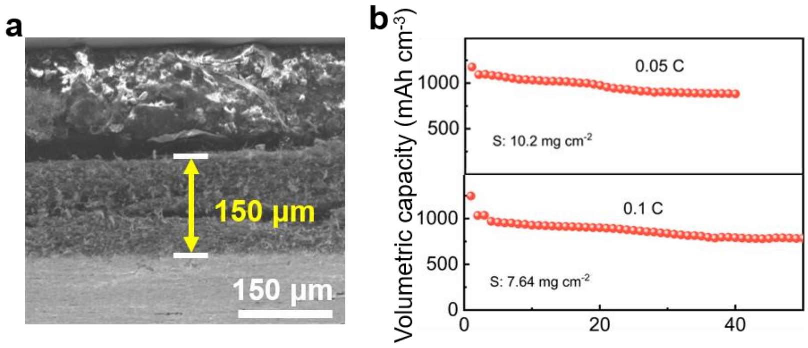

Figure S24. a) Cross-sectional SEM image of S/NiSA PCNF cathode with a sulfur loading of 10.2 mg cm^{-2}. b) Volumetric capacity of S/NiSA PCNF cathodes with a sulfur loading of 10.2 mg cm^{-2} at 0.05 C and 7.64 mg cm^{-2} at 0.1 C, respectively.

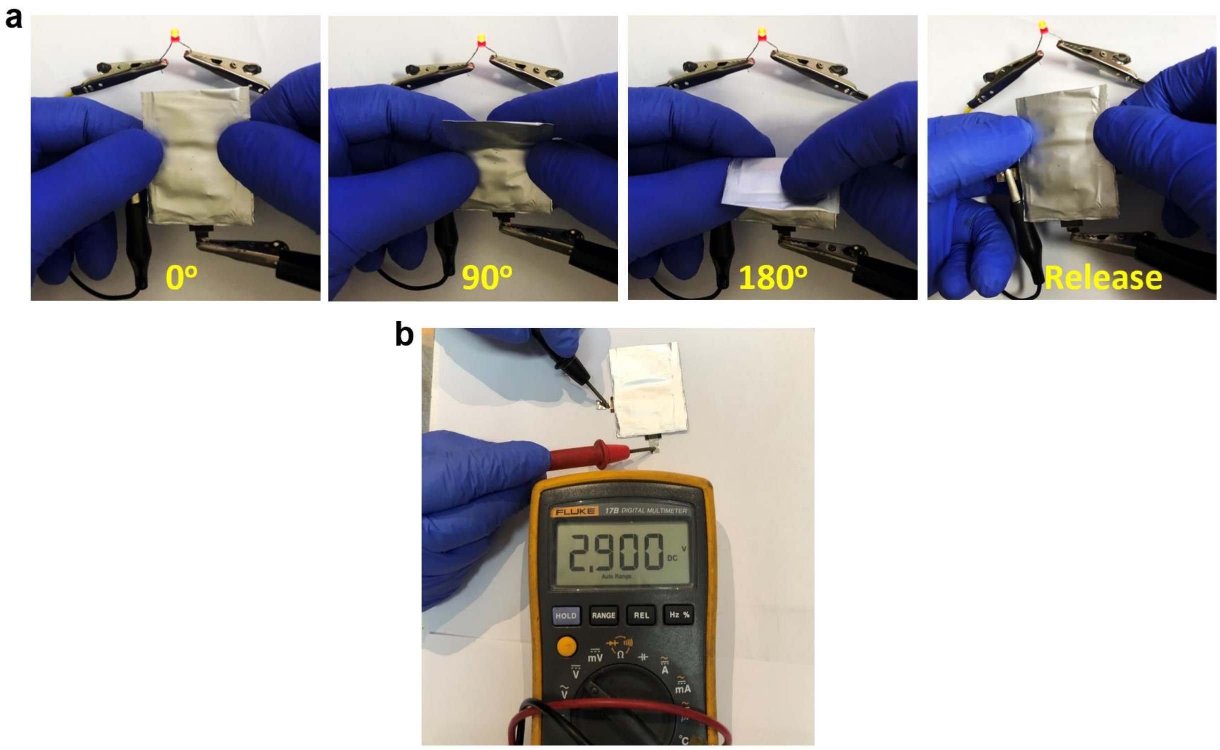

Figure S25. a) Demonstration of a flexible Li–S pouch cell to continuously power a red LED indicator. b) Open-circuit voltage of the Li–S pouch cell.

Table S1. Metal content within MSA PCNFs determined by ICP-AES.

Sample	M (at%)
NiSA PCNF	0.51
CoSA PCNF	0.43
FeSA PCNF	0.52

Table S2. Structure parameters extracted from the Ni K-edge EXAFS fitting.

Sample	Path	CN	R (Å)	σ^2 (Å^2)	R factor
NiSA PCNF	NiSA-N_4	4.56	1.86	0.01084	0.02033

Annotation: CN is the coordination number; *R* is the interatomic distance; σ^2 is the Debye-Waller factor; *R* factor is used to value the goodness of the fitting.

Table S3. The adsorption free energy (ΔG_{ads}), bond length between Li and S, S and Ni/C atom of possible bonding sites for the MN_4-doped graphene and pristine graphene (M= Fe, Co, or Ni).

system	site	ΔG_{ads} (eV)	$D_{S\text{-}M/C}$ (Å)	$D_{Li\text{-}S}$ (Å)
Fe	Fe	−3.83	2.116	2.403
	C_6	−1.86	1.891	2.417
	C_5	−2.11	1.850	2.425
Co	Co	−3.40	2.204	2.370
	C_6	−1.87	1.885	2.431
	C_5	−2.23	1.879	2.422
Ni	Ni	−2.20	2.357	2.350
	C_6	−1.75	1.886	2.428
	C_5	−1.97	1.879	2.410
Graphene	C	−1.61	1.941	2.438

Table S4. Comparison of the discharge specific capacities of NiSA PCNF/S cathodes at various current densities with those of other reported materials.

Materials	Capacity (mAh g^{-1})					Ref.
	0.2 C	0.5 C	1.0 C	2.0 C	3.0 C	
S@Fe/C_2N	1250	1085	955	856.4	774	[11]
S/Mo-N-C-4-900	1128.1	1007.3	919.8	862.2	799.4	[12]
CoSA-N-C@S	1088	978	883	829	-	[13]
S@Co–N/G	1210	942	866	790	-	[14]
Co-N-C SAC/S	1004.4	861.4	803.6	744.6	-	[15]
Li_2S-NC:SAFe	1447.0	1241.0	964	901	701.0	[16]
Ni@NG	~1420	~1350	~1180	~1060		[17]
S-SAV@NG	1230	~840	~750	~680	645	[18]
SAFe@g-C_3N_4	1255	1162	1053	926	-	[19]
Fe-N_2/CN@S	1301	1075	940	812	-	[20]
S/NiSA PCNF	1505.5	1393.2	1229	1077.5	940.4	This work

Supporting References

[1] J. F. b. G. Kresse av *Comput. Mater. Sci.* **1996**, *6*, 15.

[2] P. E. Blochl, *Phys. Rev. B Condens. Matter* **1994**, *50*, 17953.

[3] K. B. John P. Perdew, Matthias Ernzerhof, *Phys. Rev. Lett.* **1996**, *77*, 3865.

[4] S. N. Steinmann, C. Corminboeuf, *J. Chem. Theory Comput.* **2011**, *7*, 3567.

[5] K. Mathew, R. Sundararaman, K. Letchworth-Weaver, T. A. Arias, R. G. Hennig, *J. Chem. Phys.* **2014**, *140*, 084106.

[6] M. Yu, D. R. Trinkle, *J. Chem. Phys.* **2011**, *134*, 064111.

[7] S. Maintz, V. L. Deringer, A. L. Tchougreeff, R. Dronskowski, *J. Comput. Chem.* **2013**, *34*, 2557.

[8] H. Park, H. S. Koh, D. J. Siegel, *J. Phys. Chem. C* **2015**, *119*, 4675.

[9] V. Wang, N. Xu, J.-C. Liu, G. Tang, W.-T. Geng, *Comput. Phys. Commun.* **2021**, *267*, 108033.

[10] T. Pilati, *J. Appl. Cryst.* **1998**, *31*, 503.

[11] Z. Liang, D. Yang, P. Tang, C. Zhang, J. Jacas Biendicho, Y. Zhang, J. Llorca, X. Wang, J. Li, M. Heggen, J. David, R. E. Dunin‐Borkowski, Y. Zhou, J. R. Morante, A. Cabot, J. Arbiol, *Adv. Energy Mater.* **2020**, *11*, 2003507.

[12] F. Ma, Y. Wan, X. Wang, X. Wang, J. Liang, Z. Miao, T. Wang, C. Ma, G. Lu, J. Han, Y. Huang, Q. Li, *ACS Nano* **2020**, *14*, 10115.

[13] Y. Li, J. Wu, B. Zhang, W. Wang, G. Zhang, Z. W. Seh, N. Zhang, J. Sun, L. Huang, J. Jiang, J. Zhou, Y. Sun, *Energy Storage Mater.* **2020**, *30*, 250.

[14] Z. Du, X. Chen, W. Hu, C. Chuang, S. Xie, A. Hu, W. Yan, X. Kong, X. Wu, H. Ji, L. J. Wan, *J. Am. Chem. Soc.* **2019**, *141*, 3977.

[15] X. Wang, J. Sun, T. Li, Z. Song, D. Wu, B. Zhao, K. Xiang, W. Ai, X.-Z. Fu, J.-L. Luo, *Energy Storage Mater.* **2021**, *36*, 409.

[16] J. Wang, L. Jia, J. Zhong, Q. Xiao, C. Wang, K. Zang, H. Liu, H. Zheng, J. Luo, J. Yang, H. Fan, W. Duan, Y. Wu, H. Lin, Y. Zhang, *Energy Storage Mater.* **2019**, *18*, 246.

[17] L. Zhang, D. Liu, Z. Muhammad, F. Wan, W. Xie, Y. Wang, L. Song, Z. Niu, J. Chen, *Adv. Mater.* **2019**, *31*, 1903955.

[18] G. Zhou, S. Zhao, T. Wang, S. Z. Yang, B. Johannessen, H. Chen, C. Liu, Y. Ye, Y. Wu, Y. Peng, C. Liu, S. P. Jiang, Q. Zhang, Y. Cui, *Nano Lett.* **2020**, *20*, 1252.

[19] C. Lu, Y. Chen, Y. Yang, X. Chen, *Nano Lett.* **2020**, *20*, 5522.

[20] Y. Qiu, L. Fan, M. Wang, X. Yin, X. Wu, X. Sun, D. Tian, B. Guan, D. Tang, N. Zhang, *ACS Nano* **2020**, *14*, 16105.
